# Evidence for causal effects of polycystic ovary syndrome on oxidative stress: a two-sample mendelian randomisation study

**DOI:** 10.1186/s12920-023-01581-0

**Published:** 2023-06-19

**Authors:** Pu Yifu

**Affiliations:** Laboratory of Genetic Disease and Perinatal Medicine, Key Laboratory of Birth Defects and Related Diseases of Women and Children, Ministry of Education, West China Second University Hospital, Sichuan University, Chengdu, Sichuan Province 610041 China

**Keywords:** Oxidative stress, Polycystic ovary syndrome, Mendelian randomisation study

## Abstract

**Background:**

Polycystic ovary syndrome (PCOS) is often accompanied by increased oxidative stress levels; however, it is still unclear whether PCOS itself is causally related to oxidative stress (OS), whether OS can increase the occurrence of PCOS, and which characteristics of PCOS increase OS levels. Therefore, this study explored the causal relationship between PCOS, its characteristics, and OS.

**Methods:**

Two-sample bidirectional and two-sample Mendelian randomisation studies were performed based on publicly available statistics from genome-wide association studies. PCOS; its characteristics, such as testosterone, low-density lipoprotein, high-density lipoprotein; and 11 major OS markers (superoxide dismutase, glutathione S-transferase, glutathione peroxidase, catalase, uric acid, zinc, tocopherol, ascorbic acid, retinol, albumin, and total bilirubin), were studied. The main analytical method used was inverse variance weighting (IVW). Pleiotropy was evaluated using the Mendelian randomisation-Egger intercept. Q and P values were used to assess heterogeneity.

**Results:**

There was no causal relationship between PCOS and the OS indices (all P > 0.05). There was a causal relationship between the OS index, ascorbate level, and PCOS (IVW, odds ratio: 2.112, 95% confidence interval: 1.257–3.549, P = 0.005). In addition, there was a causal relationship between testosterone, low-density lipoprotein, high-density lipoprotein, sex hormone-binding globulin, body mass index, triacylglycerol, age at menarche, and most OS indices according to the IVW method. The F statistics showed that there was no weak instrumental variable. A sensitivity analysis was performed using the leave-one-out method. No pleiotropy was observed. The results were robust, and the conclusions were reliable.

**Conclusions:**

This study showed for the first time that there was no causal relationship between PCOS and OS. However, there was a causal relationship between the OS index, ascorbate level, and PCOS. It revealed that PCOS itself could not increase OS, and the increase in OS in PCOS was related to other potential factors, such as testosterone, low-density lipoprotein, high-density lipoprotein, sex hormone-binding globulin, body mass index, triacylglycerol, and age at menarche.

**Supplementary Information:**

The online version contains supplementary material available at 10.1186/s12920-023-01581-0.

## Background

Oxidative stress (OS) refers to an imbalance between the oxidative and antioxidant systems in the body [1, 2]. Common biomarkers of OS damage include enzymes, such as superoxide dismutase (SOD), glutathione S-transferase (GST), glutathione peroxidase (GPX), and catalase (CAT), and non-enzymes, such as uric acid (UA), zinc, tocopherol, ascorbic acid, retinol, albumin, and total bilirubin (TBIL) [3–5]. A balanced OS system is essential for maintaining normal body functions. Increased OS can lead to oocyte ageing and can affect the development of polycystic ovary syndrome (PCOS) and other female reproductive system diseases [6].

PCOS is one of the most common endocrine diseases in women of reproductive age [7]. In PCOS, OS levels are often increased [1, 8]. Serum malondialdehyde (MDA) levels, total oxidant status (TOS) and OS index (OSI) were reported to be higher in patients with PCOS than in the control group. Compared with the non-hyperandrogenism-PCOS subgroup, the hyperandrogenism-PCOS subgroup had higher levels of serum MDA, TOS, and OSI [9, 10], and more severe impairment of the antioxidant function of high-density lipoproteins [11]. Increasing circulating androgen levels can sensitise leukocytes, increase the expression of glucose-induced NADPH oxidase and production of oxidation-active molecules, and promote the occurrence of OS [12, 13]. Compared with non-obese patients with PCOS, patients with obesity and PCOS had higher TOS levels; however, there were no significant differences in OSI and MDA levels [9, 10]. The severity of OS was positively correlated with the hirsutism score, androgen level, blood glucose, and lipid levels [9–11].

Several oxidative stress-related enzyme gene variants included platelet-activating factor acetyl hydrolase (*PAF-AH*) G994→T and paraoxonase (*PON*) 1 Q192→R, superoxide dismutase 2 (*SOD2*) V16→A, glutathione peroxidase 1 (*GPX1*) P198→L, myeloperoxidase (*MPO*) G-463→A, cytochrome P450 2E1 (*CYP2E1*) C-1054→T variants are genetic risk factors for PCOS [14–19]. The *GCLC* gene C-129→T variant is a protective factor for the development of hyperandrogenism-PCOS [20]. These studies indicate that patients with PCOS have increased genetic susceptibility to OS and that patients with hyperandrogenism-PCOS have more severe OS than those without hyperandrogenism-PCOS. However, whether PCOS can lead to increased OS and whether OS can increase the occurrence of PCOS remain unknown. Additionally, observational studies often include potential confounding factors and reverse causality; therefore, no clear causal relationship can be obtained [21, 22].

Mendelian randomisation (MR) is an instrumental variable (IV) analysis that detects and quantifies causality using genetic variation as an IV [23]. Because of its ability to overcome potential confounding factors and reverse causality, MR has been increasingly used in observational studies in recent years [24–26]. Therefore, this study aimed to clarify the causal association between PCOS, its characteristics, and OS using a two-sample MR study.

## Methods

### Study design

**Fig. 1:**
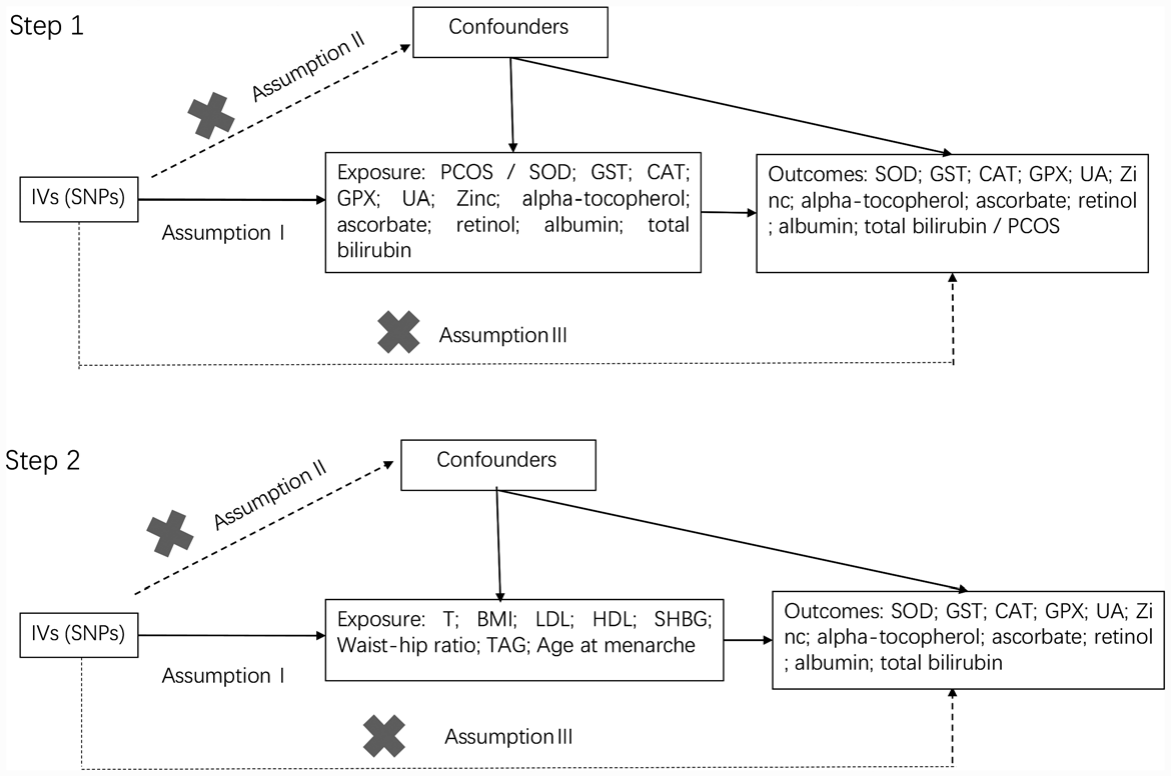
Flow chart of the Two-sample MR study design. Step 1, A two-sample bidirectional Mendelian randomisation study for PCOS and 11 oxidative stress indices; Step 2, Some two-sample Mendelian randomisation studies for characteristics indices of PCOS and 11 oxidative stress indices. IVs, instrumental variables; PCOS, polycystic ovary syndrome; GST, glutathione S-transferase; CAT, catalase; SOD, superoxide dismutase; GPX, glutathione peroxidase; UA, uric acid; SNP, single nucleotide polymorphism;T, testosterone; LDL, low-density lipoprotein; HDL, high-density lipoprotein; SHBG, sex hormone-binding globulin; BMI, body mass index; TAG, triacylglycerol

Two-sample MR design was used to detect the causal effects of PCOS and 11 OS injury biomarkers and the characteristic indices of PCOS and 11 OS indices (Fig. 1). It was based on the three hypotheses of MR: (1) Single nucleotide polymorphisms (SNPs) from genome-wide association studies (GWAS) were used as IVs, and the selected IVs were strongly correlated with exposure ; (2) IVs were not associated with confounding factors; (3) IVs affected outcomes (11 OS markers/PCOS/11 OS markers) only by exposure (PCOS/11 OS markers/ characteristic indices of PCOS) [27].

### Selection of GWAS and IVs

The GWAS of PCOS included 10,074 PCOS cases and 103,164 controls, all of whom were of European descent [28]. Fourteen independent SNPs were used according to a previous article [29]. The GWAS sources of 11 OS markers, which consisted of SOD, GST, GPX, CAT, UA, zinc, alpha-tocopherol, ascorbate, retinol, albumin, and TBIL, were used according to the previously published article [30], and the details are shown in Table 1. The participants were of European descent. The criterions of selection of IVs related to exposures were as follows (unless otherwise stated): independent SNPs (r^2^ < 0.001 and clumping distance > 10,000 kb); P value < 5 × 10^− 8^; the F statistics of all SNPs included in the MR analysis were evaluated using mRnd (an online tool named, https://shiny.cnsgenomics.com/mRnd/), all the F statistics of the included SNPs were more than 10.

**Table 1:** The GWAS sources of oxidative stress markers

Oxidative stress markers	Ancestry	Participants	SNP	Year	GWAS ID	PMID
GST	European	3301	10,534,735	2018	prot-a-1283	29875488
SOD	European	3301	10,534,735	2018	prot-a-2800	29875488
GPX	European	3301	10,534,735	2018	prot-a-1265	29875488
CAT	European	3301	10,534,735	2018	prot-a-367	29875488
UA	European	343,836	13,585,994	2018	ukb-d-30880_raw	-
alpha-tocopherol	European	6266	2,544,979	2014	met-a-571	24816252
ascorbate	European	2630	9,851,867	2018	ukb-b-19390	-
zinc	European	64,979	2,543,610	2013	ieu-a-1079	23720494
retinol	European	62,911	9,851,867	2018	ukb-b-17406	-
albumin	European	115,060	12,321,875	2020	met-d-Albumin	-
TBIL	European	342,829	13,585,986	2018	ukb-d-30840_raw	-

SNP, single nucleotide polymorphism; GWAS, genome-wide association studies; PMID, PubMed identity document; GST, glutathione S-transferase; SOD, superoxide dismutase; GPX, glutathione peroxidase; CAT, catalase; UA, uric acid; TBIL, albumin and total bilirubin

### Statistical analysis

Random effects inverse variance weighting (IVW) was used as the main analytical method to evaluate the causal relationships among PCOS, characteristic indices of PCOS, and OS. MR-Egger, weighted median, simple mode, and weighted mode were used to verify the association. Then, the MR-Egger intercept and P values were used to evaluate horizontal and vertical pleiotropy. The MR-Egger and IVW Q and P values were used to evaluate the heterogeneity. Funnel plots were constructed to determine the presence of outlier SNPs. Odds ratios (ORs) and 95% confidence intervals (CIs) were used to express the causal effects of PCOS on the OS injury biomarkers, characteristic indices of PCOS, and OS indices. All analyses were performed using the R software (version 4.2.1) two-sample MR package. A P value of less than 0.05 was considered as evidence of statistically significant causality.

## Results

### Causal association between PCOS and various OS markers: based on IVW method

As shown in Table 2, PCOS did not show a causal relationship with the 11 OS indices (based on different IVs, OR values, and 95% CI; all P values were > 0.05). Detailed information on PCOS IVs is provided in the Supplementary Materials: PCOS IVs (14SNPs). For alpha-tocopherol, nine SNPs served as IVs because the nine SNPs were found in the outcome (rs2349415, rs2178575, rs11031005, rs1784692, rs1795379, rs13164856, rs2271194, rs9696009, rs804279). For zinc, the nine SNPs (rs2349415, rs2178575, rs1795379,, rs1784692, rs13164856, rs2271194, rs804279, rs11031005, rs9696009) were also found in the outcome. When data of PCOS and zinc was harmonised, rs2271194 and rs804279 were removed as palindromic variants with intermediate allele frequencies. Therefore, seven SNPs served as IVs.

**Table 2:** Causal association between PCOS and various oxidative stress markers: based on IVW method

Oxidative stress markers	IVs (n SNPs)	Beta	SE	P	OR	95%CI
GST	13	0.007	0.076	0.932	1.007	0.867, 1.169
SOD	13	-0.015	0.068	0.828	0.985	0.863, 1.125
GPX	13	0.014	0.093	0.879	1.014	0.845, 1.218
CAT	13	0.004	0.068	0.958	1.004	0.879, 1.146
UA	13	1.105	0.748	0.140	3.018	0.696, 13.087
alpha-tocopherol	9	-0.022	0.016	0.188	0.979	0.948, 1.011
ascorbate	13	-0.013	0.015	0.397	0.987	0.959, 1.017
zinc	7	-0.008	0.096	0.934	0.992	0.821, 1.198
retinol	13	0.025	0.015	0.102	1.025	0.995, 1.056
albumin	13	0.020	0.012	0.093	1.020	0.997, 1.044
TBIL	13	-0.013	0.034	0.709	0.987	0.923, 1.056

PCOS, polycystic ovary syndrome; IVW, inverse variance weighting; SOD, superoxide dismutase; GST, glutathione S-transferase, GPX, glutathione peroxidase, CAT, catalase, UA, uric acid, TBIL, total bilirubin; SNP, Single Nucleotide polymorphisms; IVs, instrumental variables; OR, Odds ratio; CI, confidence interval; SE, standard error; n, number

### Causal association between PCOS and OS markers: heterogeneity and pleiotropy

As shown in Table 3, there was no pleiotropy according to the MR-Egger intercept and P value. Meanwhile, there was no heterogeneity except for GPX and UA.

**Table 3:** Causal association between PCOS and oxidative stress markers: heterogeneity and pleiotropy

Oxidative stress markers	heterogeneity				pleiotropy	
	MR Egger		IVW			
	Q	P value	Q	P value	MR egger intercept	P value
GST	13.686	0.251	15.245	0.228	-0.045	0.287
SOD	7.163	0.786	9.034	0.700	0.050	0.199
GPX	21.564	0.028	22.815	0.029	0.041	0.441
CAT	10.531	0.483	11.337	0.500	-0.033	0.389
UA	32.404	0.001	33.727	0.001	0.275	0.516
Zinc	5.016	0.414	5.184	0.520	-0.021	0.699
alpha-tocopherol	1.113	0.993	1.116	0.997	0.000	0.960
ascorbate	10.554	0.481	11.025	0.527	-0.005	0.507
retinol	8.777	0.642	9.400	0.668	-0.006	0.447
albumin	13.207	0.280	13.374	0.342	0.002	0.716
TBIL	17.555	0.092	17.916	0.118	0.009	0.644

PCOS, polycystic ovary syndrome; IVW, inverse variance weighting; SOD, superoxide dismutase; GST, glutathione S-transferase, GPX, glutathione peroxidase, CAT, catalase, UA, uric acid, TBIL, total bilirubin

### Causal association between PCOS and SOD according to five methods

**Fig. 2:**
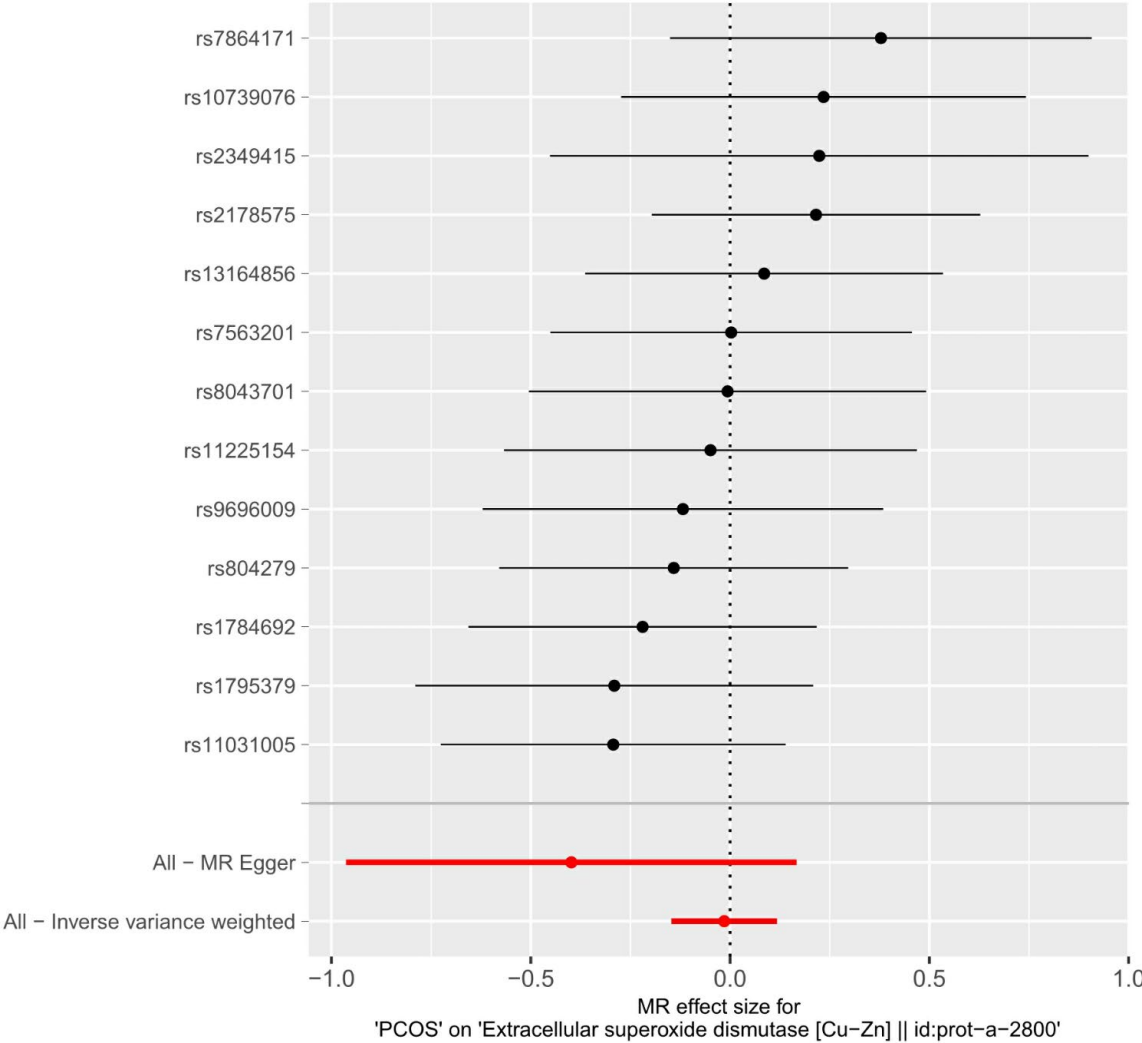
MR effect size for PCOS on SOD PCOS, polycystic ovary syndrome; SOD, superoxide dismutase

**Fig. 3:**
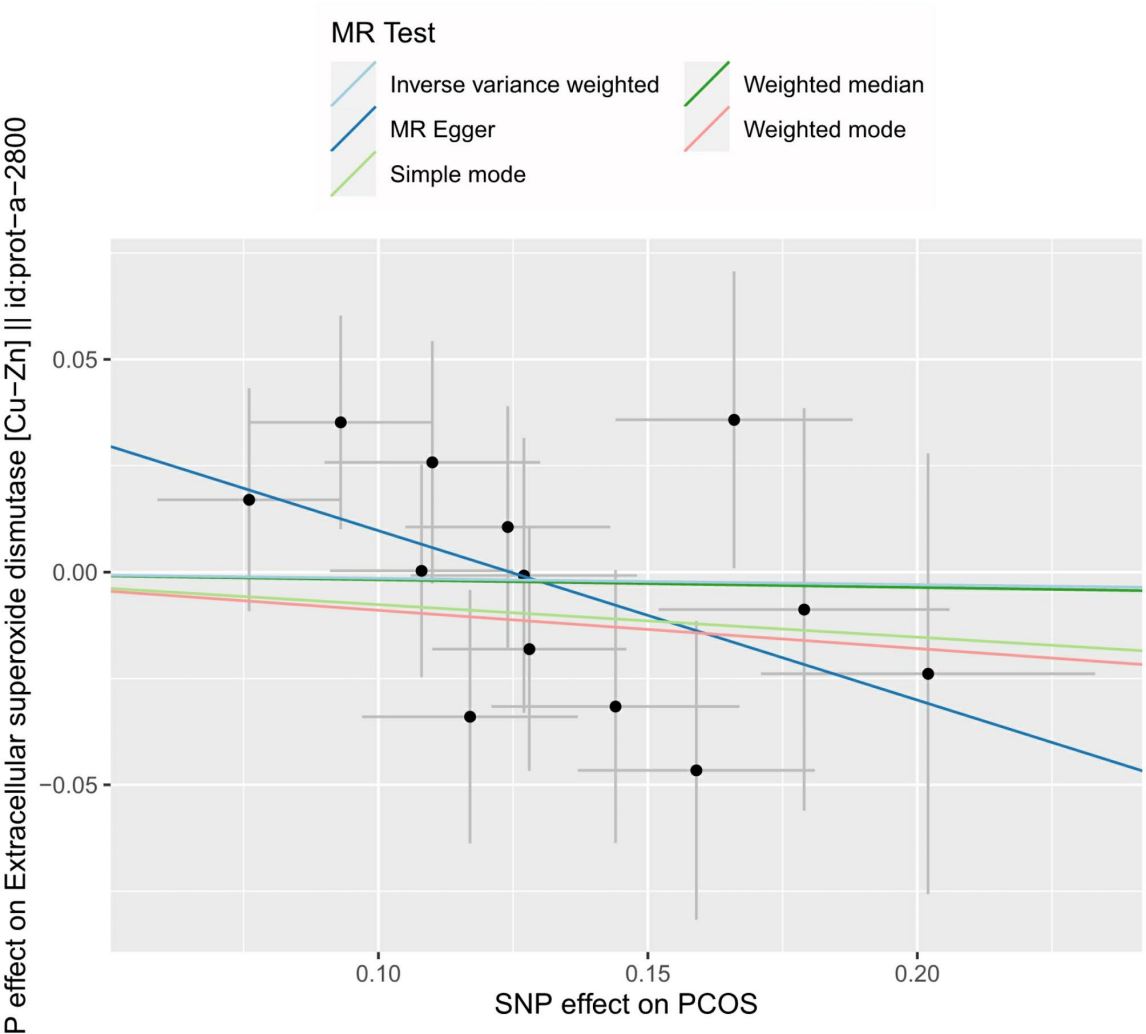
Scatter plot of the MR analysis of PCOS on SOD PCOS, polycystic ovary syndrome; SOD, superoxide dismutase

**Fig. 4:**
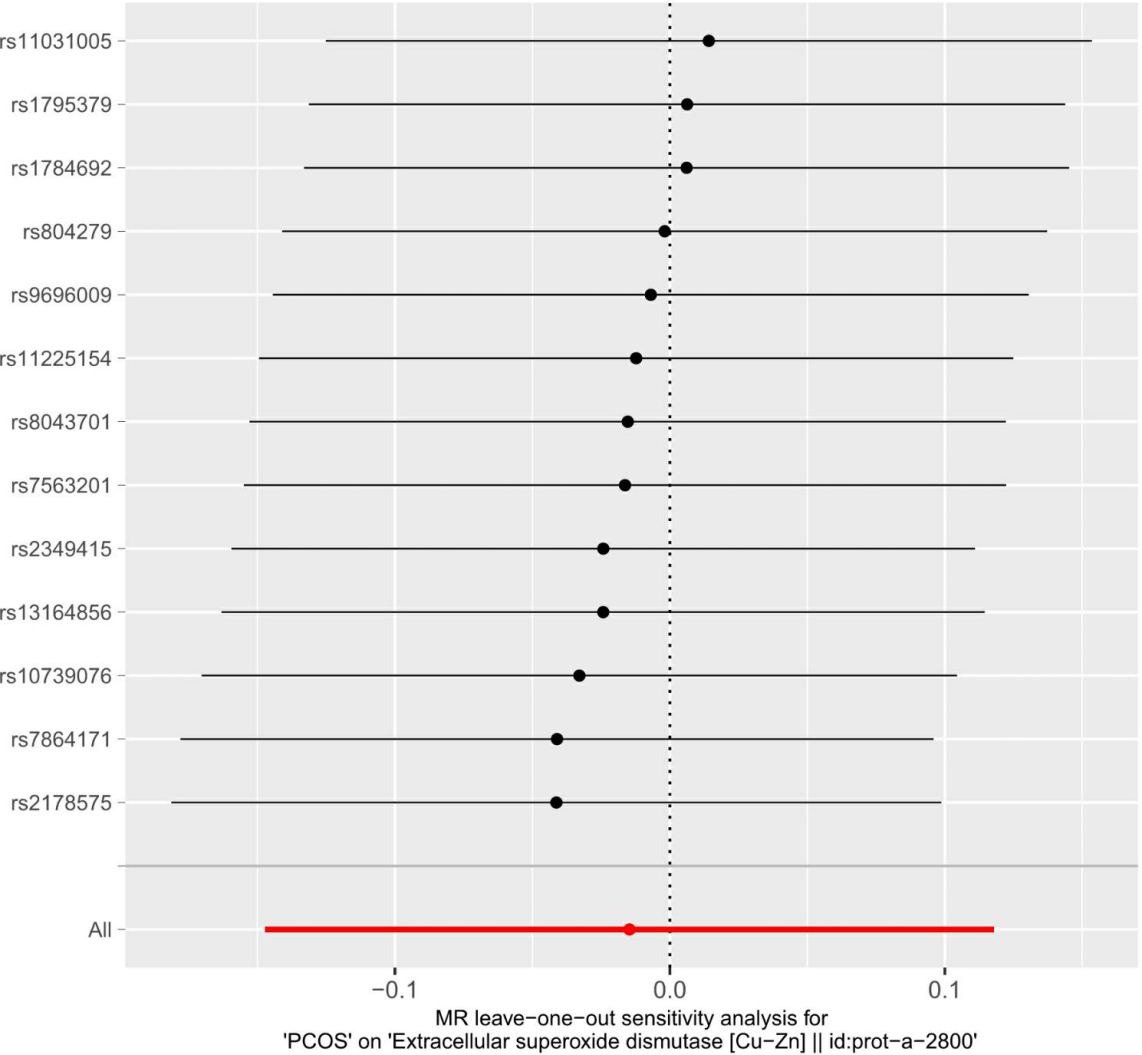
Leave-one-out regression analysis of PCOS on SOD PCOS, polycystic ovary syndrome; SOD, superoxide dismutase

**Fig. 5:**
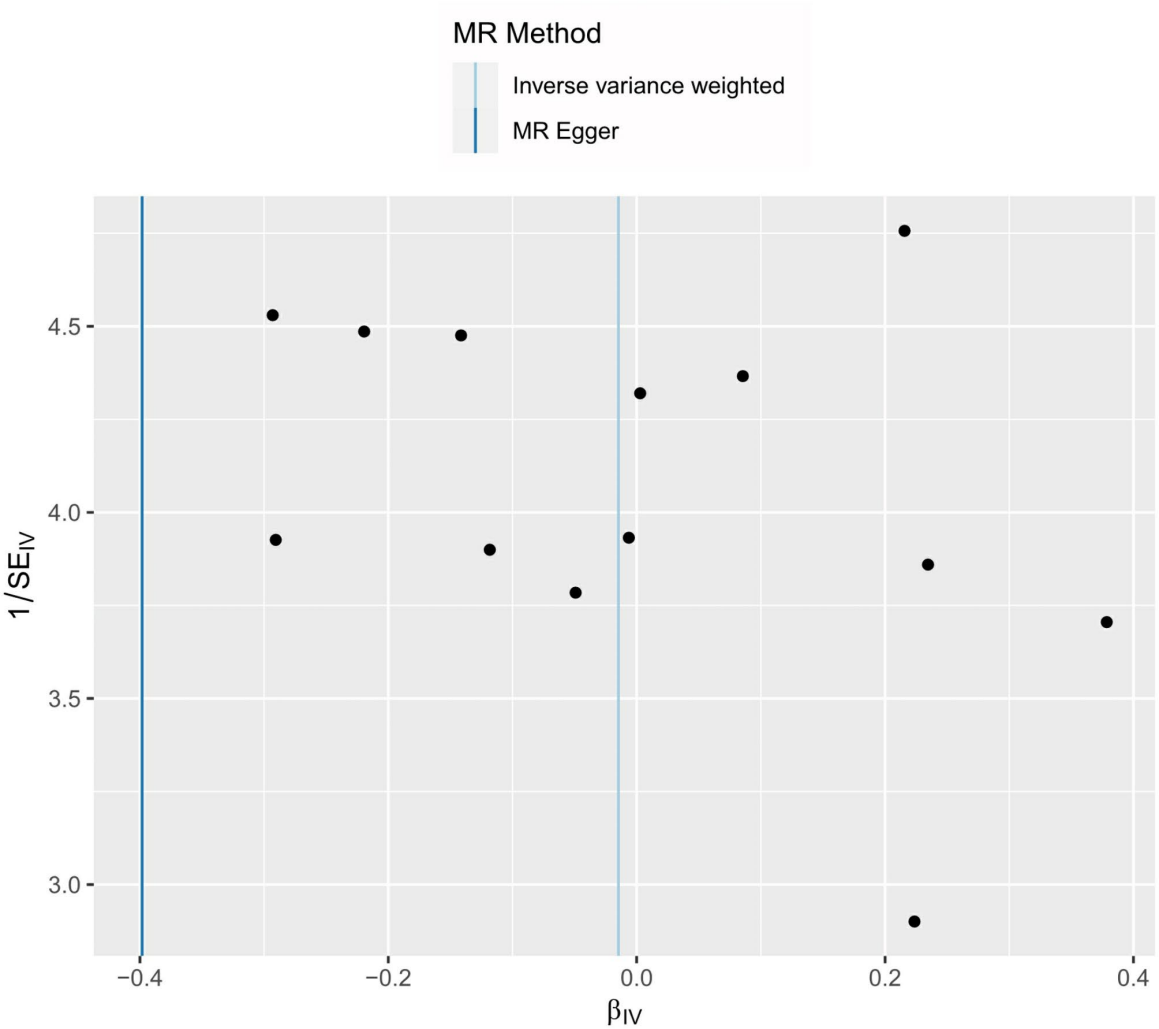
Funnel plot of the MR analysis of PCOS on SOD PCOS, polycystic ovary syndrome; SOD, superoxide dismutase

As shown in Table 4, PCOS did not show a causal relationship with SOD according to the five methods. MR sizes for PCOS on SOD, scatter plots, leave-one-out, and funnel plots are shown in Figs. 2, 3 and 4, and 5, respectively.

**Table 4:** Causal association between PCOS and SOD.

Methods	IVs (n SNPs)	Beta	SE	P	OR	95%CI
MR Egger	13	-0.398	0.288	0.195	0.672	0.382, 1.182
Weighted median	13	-0.018	0.091	0.842	0.982	0.822, 1.173
Inverse variance weighted	13	-0.015	0.068	0.828	0.985	0.863, 1.125
Simple mode	13	-0.076	0.164	0.650	0.926	0.671, 1.278
Weighted mode	13	-0.090	0.162	0.590	0.914	0.665, 1.256

PCOS, polycystic ovary syndrome; SOD, superoxide dismutase; SNP, Single Nucleotide polymorphisms; IVs, instrumental variables; OR, Odds ratio; CI, confidence interval; SE, standard error; n, number

### Causal association between PCOS and GST /GPX /CAT /UA /zinc /alpha-tocopherol /ascorbic acid /retinol /albumin /TBIL according to five methods

PCOS did not show causal relationship with GST (Supplementary Materials: Table S1) /GPX (Table S2) /CAT (Table S3) /UA (Table S4) /zinc (Table S5) / alpha-tocopherol (Table S6) /ascorbic acid (Table S7) /**retinol** (Table S8) /**albumin** (Table S9) /**TBIL** (Table S10) according to five methods. The MR effect size, scatter plot, leave-one-out, and funnel plots are shown in Supplementary Materials Figure S1-4 /S5-8 /S9-12 /S13-16 /S17-20 /S21-24 /S25-28 /S29-32 /S33-36 /S37-40.

### Causal association between various OS markers and PCOS

As shown in Table 5, most OS indices did not show a causal relationship with PCOS (based on different IVs, OR values, and 95% CI; all P values were > 0.05), except for tocopherol (MR-Egger, OR: 3.74, 95% CI: 1.297–10.783, P = 0.035) and ascorbate (IVW, OR: 2.112, 95% CI: 1.257–3.549, P = 0.005).

**Table 5:** The associations between genetically predicted oxidative stress indices and the risk of PCOS.

Exposure	GWAS ID	Outcome*	n SNPs	Method	OR (95%CI)	P value
GST	prot-a-1283	PCOS	11	IVW	1.013(0.904–1.135)	0.824
			11	Weighted median	1.011(0.876–1.167)	0.880
			11	MR-Egger	1.024(0.797–1.316)	0.857
CAT	prot-a-367	PCOS	26	IVW	1.028(0.926–1.142)	0.601
			26	Weighted median	1.079(0.926–1.257)	0.330
			26	MR-Egger	1.303(0.919–1.849)	0.151
SOD	prot-a-2800	PCOS	23	IVW	1.050(0.936–1.178)	0.401
			23	Weighted median	1.010(0.860–1.187)	0.900
			23	MR-Egger	1.118(0.851–1.468)	0.431
GPX	prot-a-1265	PCOS	21	IVW	1.061(0.932–1.207)	0.371
			21	Weighted median	1.048(0.917–1.198)	0.492
			21	MR-Egger	1.044(0.811–1.344)	0.743
UA	ukb-d-30880_raw	PCOS	613	IVW	0.998(0.996–1.000)	0.115
			613	Weighted median	1.000(0.997–1.003)	0.964
			613	MR-Egger	1.000(0.996–1.003)	0.760
Tocopherol	met-a-571	PCOS	12	IVW	1.348(0.795–2.286)	0.268
			12	Weighted median	1.412(0.657–3.032)	0.377
			12	MR-Egger	3.74(1.297–10.783)	0.035^a^
Zinc	ieu-a-1079	PCOS	11	IVW	1.102(0.967–1.257)	0.144
			11	Weighted median	1.100(0.932–1.300)	0.261
			11	MR-Egger	1.408(0.927–2.139)	0.143
Ascorbate	ukb-b-19390	PCOS	23	IVW	2.112(1.257–3.549)	0.005^b^
			23	Weighted median	2.035(0.998–4.150)	0.051
			23	MR-Egger	1.846(0.474–7.184)	0.387
Retinol	ukb-b-17406	PCOS	19	IVW	0.852(0.410–1.769)	0.667
			19	Weighted median	1.031(0.441–2.411)	0.944
			19	MR-Egger	0.446(0.067–2.988)	0.417
Albumin	met-d-Albumin	PCOS	114	IVW	1.139(0.900–1.440)	0.279
			114	Weighted median	1.087(0.748–1.580)	0.660
			114	MR-Egger	0.951(0.581–1.556)	0.841
TBIL	ukb-d-30840_raw	PCOS	240	IVW	0.977(0.950–1.004)	0.096
			240	Weighted median	0.971(0.933–1.011)	0.156
			240	MR-Egger	0.970(0.939–1.001)	0.060

PCOS, polycystic ovary syndrome; GST, glutathione S-transferase; CAT, catalase; SOD, superoxide dismutase; GPX, glutathione peroxidase; UA, uric acid; TBIL, total bilirubin; SNP, single nucleotide polymorphism; GWAS, genome-wide association studies; ID, identity document; IVW, inverse variance weighted; n, number

^a^P < 0.05, Tocopherol and PCOS have the causal effect according to MR-Egger method

^b^P < 0.05, Ascorbate and PCOS have the causal effect according to IVW method

Selection of IVs related to exposures: independent SNPs (r^2^ < 0.01 and distance > 250 kb); P value < 1 × 10^− 5^; all the F statistics of the included SNPs were more than 10

*The source of PCOS GWAS is from the website-10.17863/CAM.36024, Day, F. (2019). Summary statistics for PCOS. Apollo - University of Cambridge Repository

### Causal association between various characteristics indices of PCOS and OS markers: based on IVW method

As shown in Table 6, the characteristic indices of PCOS showed a causal relationship with most OS indices (based on different IVs, OR values, and 95% CI; all P values were less than 0.05).

**Table 6:** The associations between genetically predicted characteristics indices of PCOS and the risk of oxidative stress

Exposure	GWAS ID	Outcome	GWAS ID	n SNPs	Method	OR (95%CI)	P value
T	ebi-a-GCST90012104	retinol	ukb-b-17406	92	IVW	0.929(0.872–0.990)	0.023
T	ebi-a-GCST90012114	UA	ukb-d-30880_raw	171	IVW	1.139e-5(4.415e-9–2.940e-2)	0.005
T	ebi-a-GCST90012114	TBIL	ukb-d-30840_raw	171	IVW	2.046(1.331–3.144)	0.001
LDL	ieu-b-110	retinol	ukb-b-17406	151	IVW	0.910(0.878–0.943)	2.726e-7
LDL	ieu-b-110	tocopherol	met-a-571	55	IVW	1.063(1.006–1.124)	0.031
LDL	ieu-b-110	GPX	prot-a-1265	164	IVW	1.181(1.015–1.375)	0.032
HDL	ieu-b-109	UA	ukb-d-30880_raw	340	IVW	0.000(1.542e-5–0.002)	2.843e-11
SHBG	ieu-b-4870	UA	ukb-d-30880_raw	187	IVW	7.079e-5(3.144e-6–0.002)	1.811e-9
SHBG	ieu-b-4871	UA	ukb-d-30880_raw	190	IVW	0.002(1.170e-4–0.042)	4.563e-5
SHBG	ieu-b-4871	albumin	met-d-Albumin	191	IVW	1.069(1.005–1.137)	0.033
BMI	ukb-b-19953	UA	ukb-d-30880_raw	441	IVW	1.303e9(4.571e + 7–3.713e + 10)	1.160e-34
BMI	ukb-b-19953	ascorbate	ukb-b-19390	440	IVW	0.938(0.905–0.971)	< 0.001
BMI	ukb-b-19953	retinol	ukb-b-17406	440	IVW	0.915(0.885–0.946)	1.271e-7
BMI	ukb-b-19953	albumin	met-d-Albumin	441	IVW	0.844(0.818–0.870)	2.910e-27
BMI	ukb-b-19953	TBIL	ukb-d-30840_raw	441	IVW	0.663(0.600–0.734)	1.623e-15
Waist-hip ratio	ieu-b-4830	GST	prot-a-1283	66	IVW	81.573(1.364–4.878e + 3)	0.035
TAG	ieu-b-4850	SOD	prot-a-2800	92	IVW	0.746(0.643–0.866)	0.001
TAG	ieu-b-4850	GPX	prot-a-1265	92	IVW	1.248(1.075–1.447)	0.004
Age at menarche	ieu-b-4822	CAT	prot-a-367	50	IVW	1.113(1.001–1.239)	0.048
Age at menarche	ieu-b-4822	UA	ukb-d-30880_raw	50	IVW	0.215(0.063–0.727)	0.013
Age at menarche	ieu-b-4822	albumin	met-d-Albumin	50	IVW	1.021(1.002–1.041)	0.030

PCOS, polycystic ovary syndrome; T, testosterone; LDL, low-density lipoprotein; HDL, high-density lipoprotein; SHBG, sex hormone-binding globulin; BMI, body mass index; TAG, triacylglycerol; GST, glutathione S-transferase; CAT, catalase; SOD, superoxide dismutase; GPX, glutathione peroxidase; UA, uric acid; TBIL, total bilirubin; SNP, single nucleotide polymorphism; GWAS, genome-wide association studies; ID, identity document; IVW, inverse variance weighted; n, number

## Discussion

To the best of our knowledge, this is the first study exploring the causal effects of polycystic ovary syndrome and characteristic indices of PCOS on OS. In this study, phenotypic GWAS data were analysed using two-sample MR, and no evidence of a causal relationship between PCOS and OS markers was found. However, there was a causal relationship between OS index, ascorbate, and PCOS. This revealed that PCOS itself could not increase OS, ascorbate could increase the occurrence of PCOS, and the increase in the oxidative level of PCOS was related to other potential factors, such as testosterone, low-density lipoprotein, high-density lipoprotein, sex hormone-binding globulin, body mass index, triacylglycerol, and age at menarche, which may act as characteristic indices of PCOS. An observational study has emphasised the association between PCOS and OS [31]. However, relevant MR studies regarding this association are lacking. In addition to observational studies, relevant mechanistic studies have been conducted on OS and PCOS. A study pointed out that OS contributed to insulin resistance in the skeletal muscles of mice with dehydroepiandrosterone-induced PCOS [32]. Salidroside alleviates OS and apoptosis via AMPK/Nrf2 pathway in dihydrotestosterone-induced human granulosa cell line KGN [33].

A meta-analysis has indicated that circulating markers of OS are abnormal in patients with PCOS [1]. OS in patients with PCOS may be associated with several diseases [34, 35]. A few antioxidants can ameliorate PCOS through reducing OS, such as Tempol [36], Kelulut honey [37], Standardised Aronia melanocarpa [38], astaxanthin [39], resveratrol [40], and N-acetyl cysteine [41]. Besides, silibinin [42] and vitamin E supplementation [43] as well as melatonin and/or magnesium supplementation [44] also ameliorate PCOS by reducing the level of OS.

This study included 11 different markers of OS injury, 10 characteristic indices of PCOS, and large-sample PCOS GWAS data from the same race- European ancestor. The proposed method has several advantages. First, it included a two-sample bidirectional MR. Hence, a causal association between OS and PCOS can be proven in reverse. In addition, PCOS itself does not increase OS; therefore, characteristic indices of PCOS were used to explore the causal effects on OS. Some indices related to PCOS characteristics have causal effects on OS. PCOS is a heterogeneous endocrine disorder. Patients with PCOS often present with hyperandrogenemia, glucose and lipid metabolism disorders, obesity, waist-to-hip ratio imbalance, menstrual disorders, ovulation abnormalities, and other symptoms. This study provides evidence for the need to regulate glycaemic and lipid metabolism, control body weight, reduce hyperandrogenemia, and replenish ascorbate and tocopherol in patients with polycystic ovary syndrome, with the aim to reduce the levels of OS or the occurrence of PCOS.

Meanwhile, this study has some limitations. First, GWAS data were obtained from a European ancestor, and whether this conclusion is true for other races needs to be studied. In addition, some analyses used a small number of SNPs (less than 10), and some analyses were not pleiotropic but heterogeneous, such as GPX and UA, which may lead to inaccurate results and compromise confidence. With the continuous update and release of PCOS GWAS data [28, 45–48], we are likely to overcome these limitations. Finally, the conclusion may be more accurate if the measures of OS included only women.

## Conclusions

In summary, this two-sample MR study indicated that genetically predicted PCOS was not significantly associated with oxidative stress; however, the OS index, ascorbate, was significantly associated with PCOS. PCOS itself does not lead to an increase in OS levels, and the increase in OS levels in PCOS is related to other potential factors, such as hyperandrogenism, low-density lipoprotein, high-density lipoprotein, sex hormone-binding globulin, body mass index, triacylglycerol, and age at menarche. It is necessary to regulate glycaemic and lipid metabolism, control body weight, reduce hyperandrogenemia, and replenish ascorbate and tocopherol to reduce the levels of OS or the occurrence of PCOS. Further scientific studies are needed to uncover the mechanisms underlying the increased levels of OS in PCOS.

## Electronic supplementary material

Below is the link to the electronic supplementary material.

Supplementary Material 1:

Supplementary Material 2

Supplementary Material 3

Supplementary Material 4

Supplementary Material 5

Supplementary Material 6

Supplementary Material 7

Supplementary Material 8

Supplementary Material 9

Supplementary Material 10

Supplementary Material 11

Supplementary Material 12

Supplementary Material 13

Supplementary Material 14

Supplementary Material 15

Supplementary Material 16

Supplementary Material 17

Supplementary Material 18

Supplementary Material 19

Supplementary Material 20

Supplementary Material 21

Supplementary Material 22

Supplementary Material 23

Supplementary Material 24

Supplementary Material 25

Supplementary Material 26

Supplementary Material 27

Supplementary Material 28

Supplementary Material 29

Supplementary Material 30

Supplementary Material 31

Supplementary Material 32

Supplementary Material 33

Supplementary Material 34

Supplementary Material 35

Supplementary Material 36

Supplementary Material 37

Supplementary Material 38

Supplementary Material 39

Supplementary Material 40

Supplementary Material 41

Supplementary Material 42

Supplementary Material 43

Supplementary Material 44

Supplementary Material 45

Supplementary Material 46

Supplementary Material 47

Supplementary Material 48

Supplementary Material 49

Supplementary Material 50

Supplementary Material 51

## Data Availability

These oxidative stress injury biomarkers were based on the study-Lu Z, Pu C, Zhang Y, et al. Oxidative Stress and Psychiatric Disorders: Evidence from the Bidirectional Mendelian Randomization Study J. Antioxidants (Basel), 2022, (11). DOI:10.3390/antiox11071386. Detailed oxidative stress injury biomarkers are shown in Table 1. Detailed information on studies and datasets used in this study. PCOS IVs were based on the study-Zhu T, Cui J, Goodarzi MO. Polycystic Ovary Syndrome and Risk of Type 2 Diabetes, Coronary Heart Disease, and Stroke J. Diabetes, 2021, (70):627 − 37. Doi: 10.2337/db20-0800. Detailed PCOS IVs are shown in Table 1. PCOS SNPs were used to construct the main IV in Europeans.

## Abbreviations

PCOS: Polycystic ovary syndrome
MR: Mendelian randomisation
IVW: Inverse variance weighting
SOD: Superoxide dismutase
GST: Glutathione S-transferase
GPX: Glutathione peroxidase
CAT: Catalase
UA: Uric acid
TBIL: Total bilirubin
SNP: Single nucleotide polymorphism
IVs: Instrumental variables
OR: Odds ratio
CI: Confidence interval
SE: Standard error
n: Number
T: Testosterone
LDL: Low-density lipoprotein
HDL: High-density lipoprotein
SHBG: Sex hormone-binding globulin
BMI: Body mass index
TAG: Triacylglycerol
GWAS: Genome-wide association studies
OS: Oxidative stress
TOS: Total oxidant status
