# Supplementary figures and images for: Evidence for causal effects of polycystic ovary syndrome on oxidative stress: a two-sample mendelian randomisation study
Source: BMC Med Genomics. 2023 Jun 19;16:141. doi: 10.1186/s12920-023-01581-0 (PMC10278295; doi:10.1186/s12920-023-01581-0)

Figure S13. MR effect size for PCOS on UA


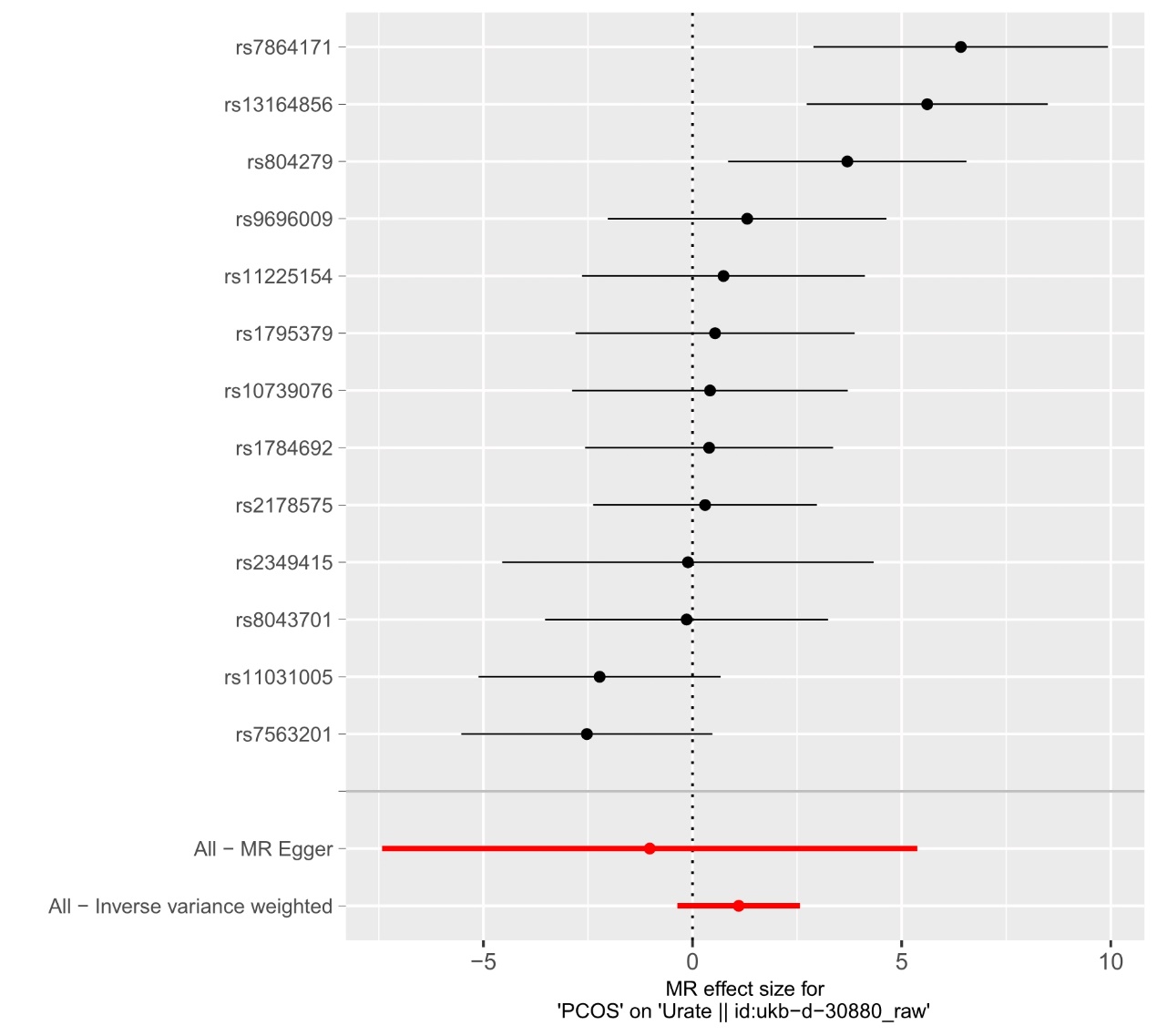


PCOS, Polycystic ovary syndrome; UA, uric acid.

Supplement: Supplementary file 5 — Supplementary Material 5 [file 12920_2023_1581_MOESM5_ESM.docx]

Figure S16. funnel plot of the MR analysis of PCOS on UA


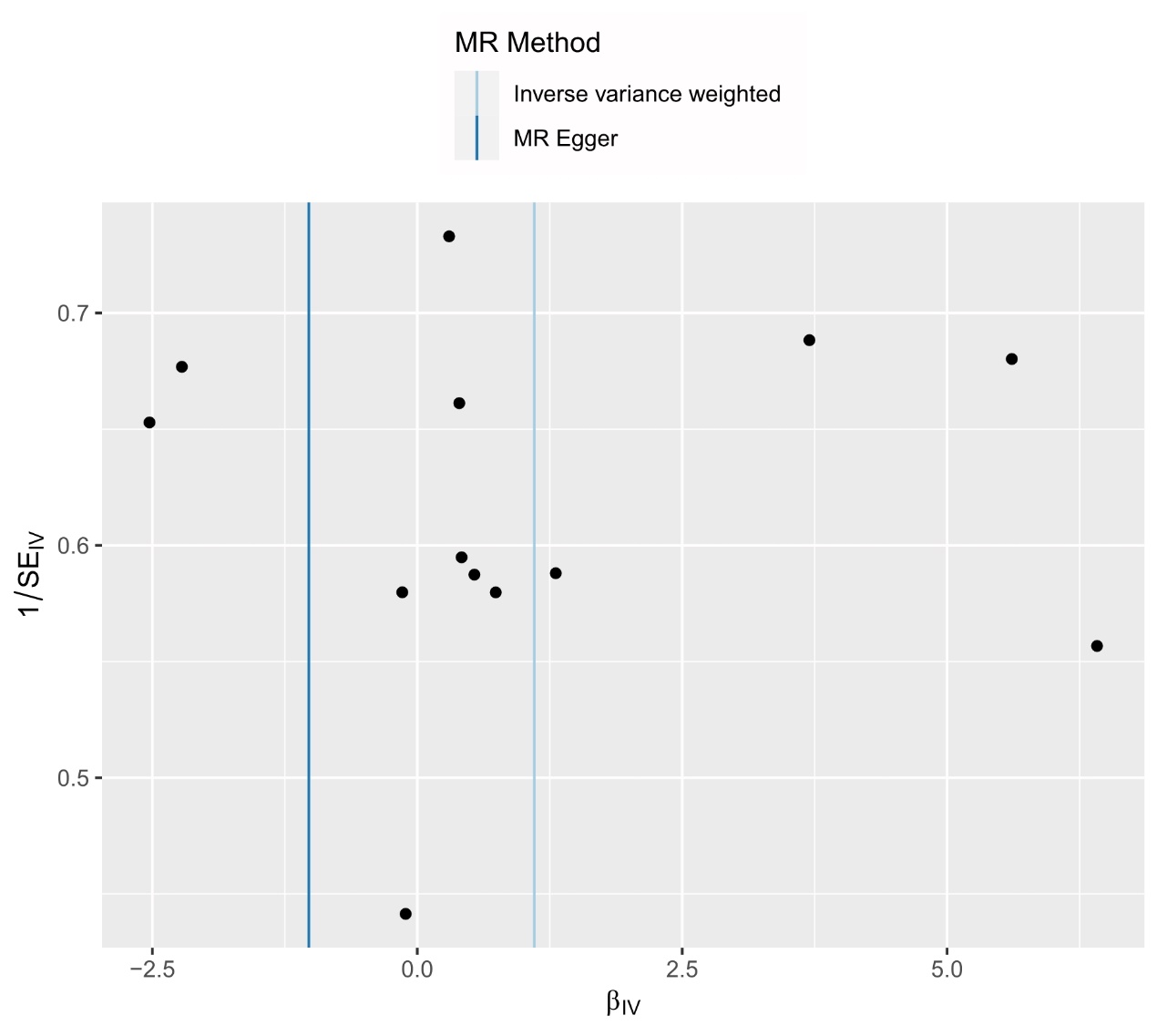


PCOS, Polycystic ovary syndrome; UA, uric acid.

Supplement: Supplementary file 8 — Supplementary Material 8 [file 12920_2023_1581_MOESM8_ESM.docx]

Figure S17. MR effect size for PCOS on Zinc


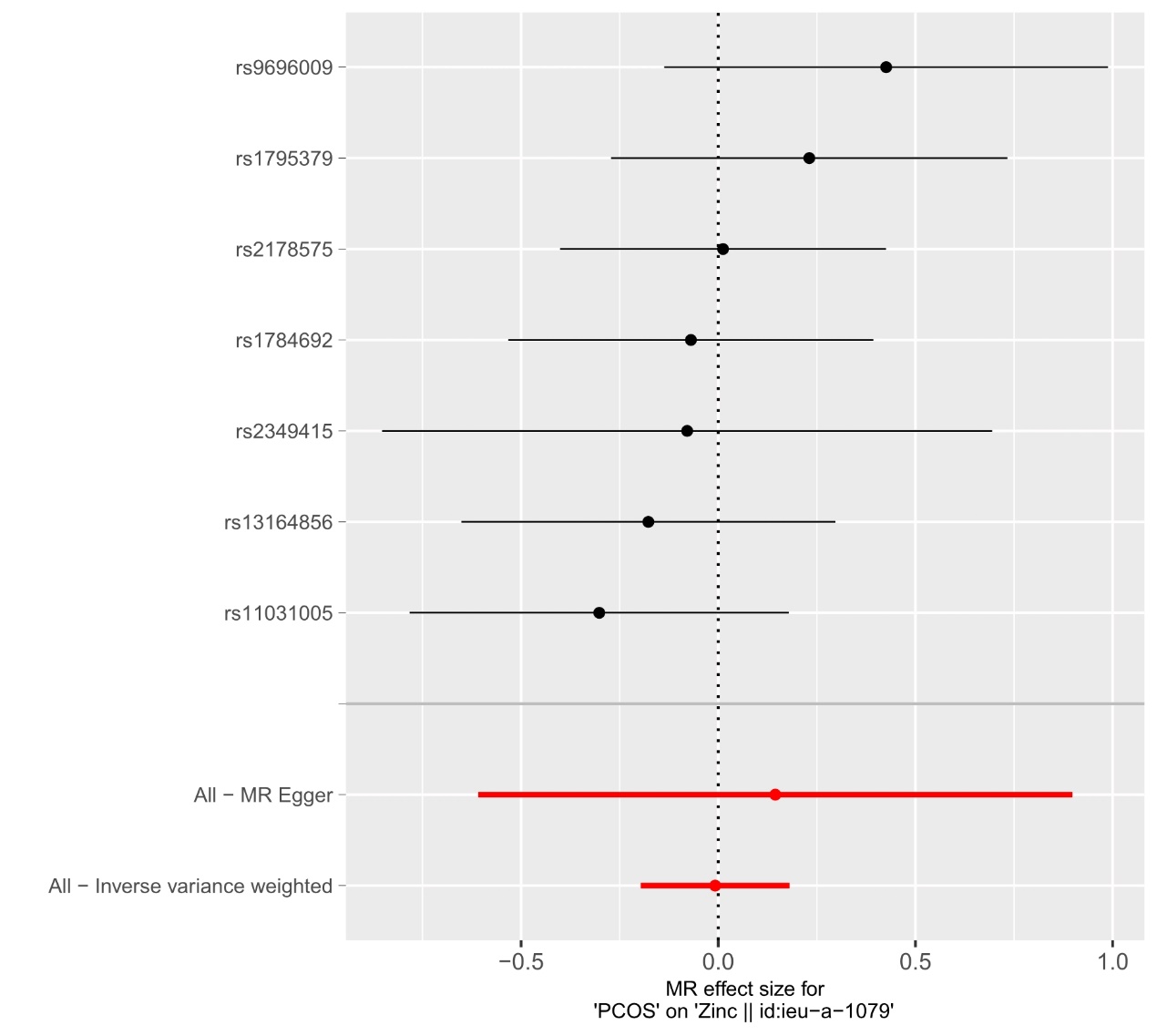


PCOS, Polycystic ovary syndrome.

Supplement: Supplementary file 9 — Supplementary Material 9 [file 12920_2023_1581_MOESM9_ESM.docx]

Figure S18. scatter plot of the MR analysis of PCOS on Zinc


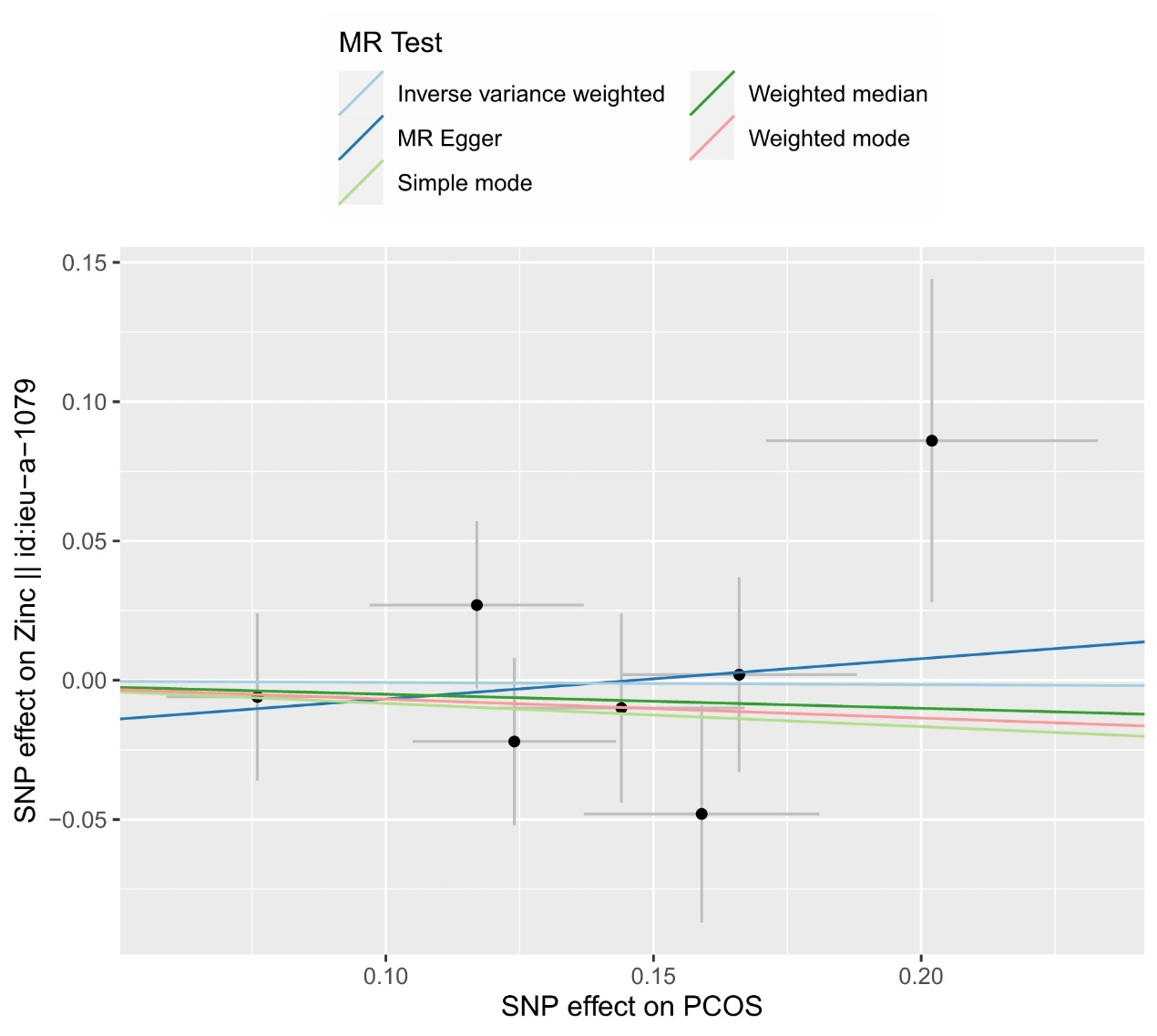


PCOS, Polycystic ovary syndrome.

Supplement: Supplementary file 10 — Supplementary Material 10 [file 12920_2023_1581_MOESM10_ESM.docx]

Figure S19. leave-one-out regression analysis of PCOS on Zinc


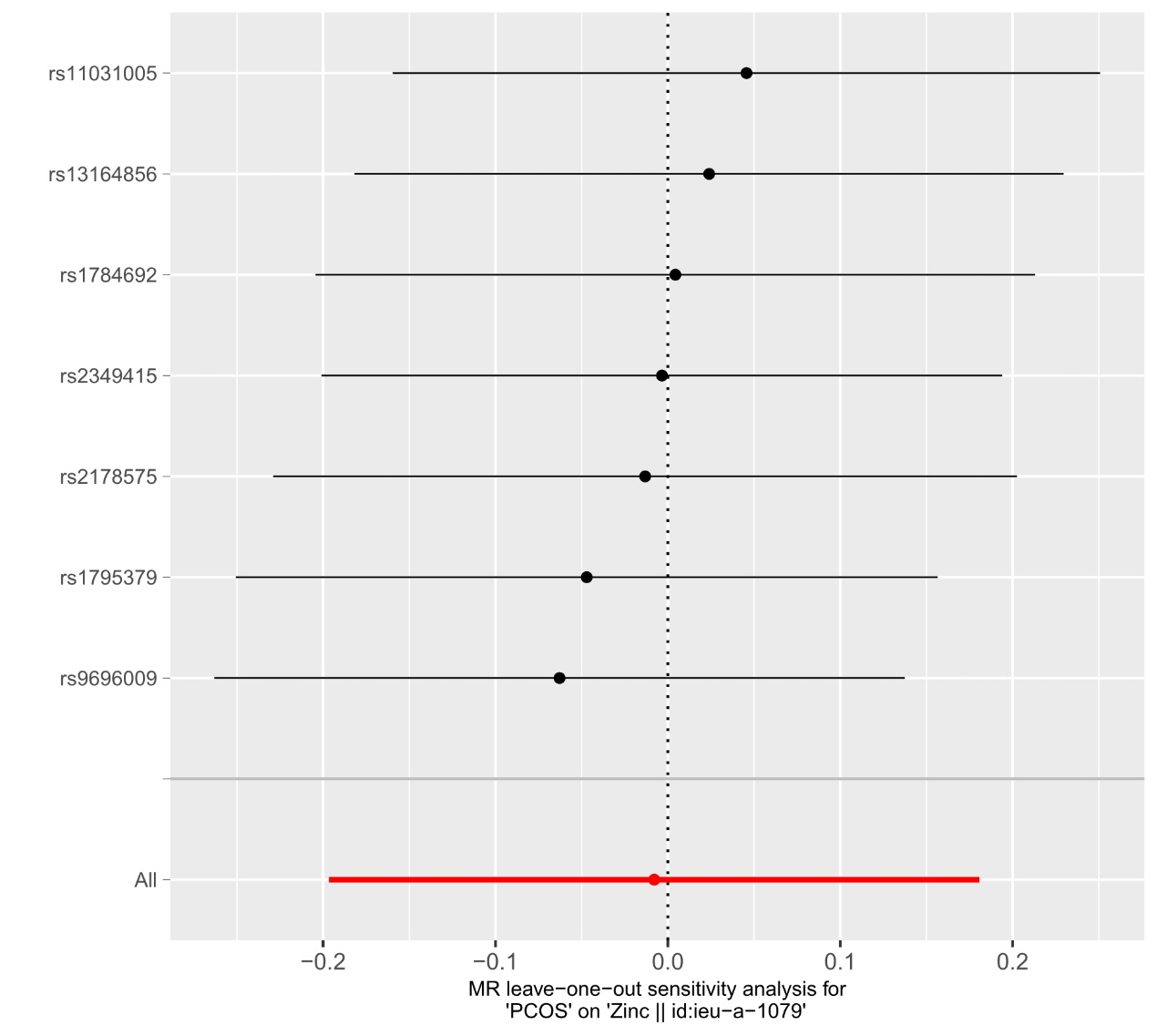


PCOS, Polycystic ovary syndrome.

Supplement: Supplementary file 11 — Supplementary Material 11 [file 12920_2023_1581_MOESM11_ESM.docx]

Figure S20. funnel plot of the MR analysis of PCOS on Zinc


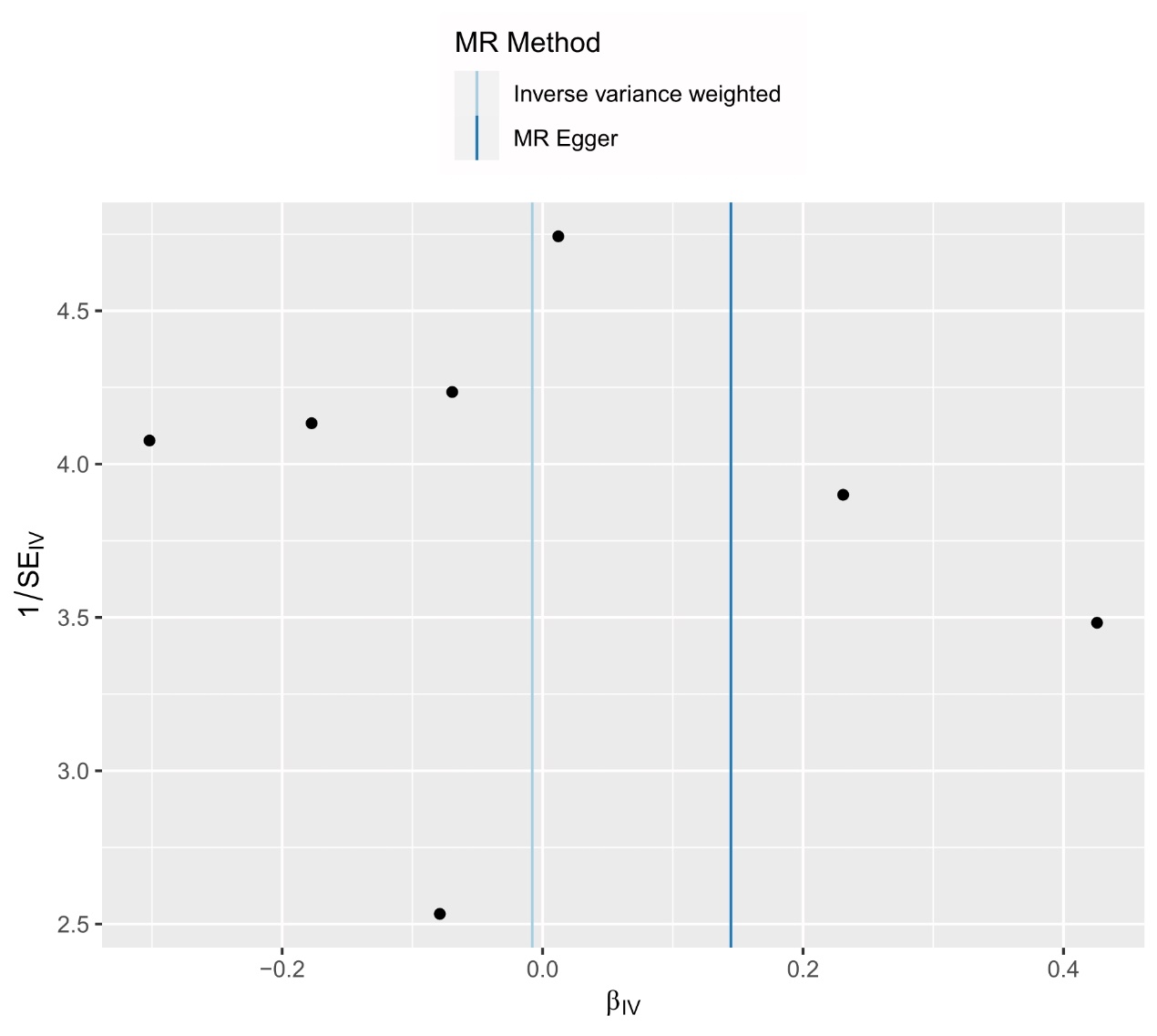


PCOS, Polycystic ovary syndrome.

Supplement: Supplementary file 13 — Supplementary Material 13 [file 12920_2023_1581_MOESM13_ESM.docx]

Figure S21. MR effect size for PCOS on alpha-tocopherol


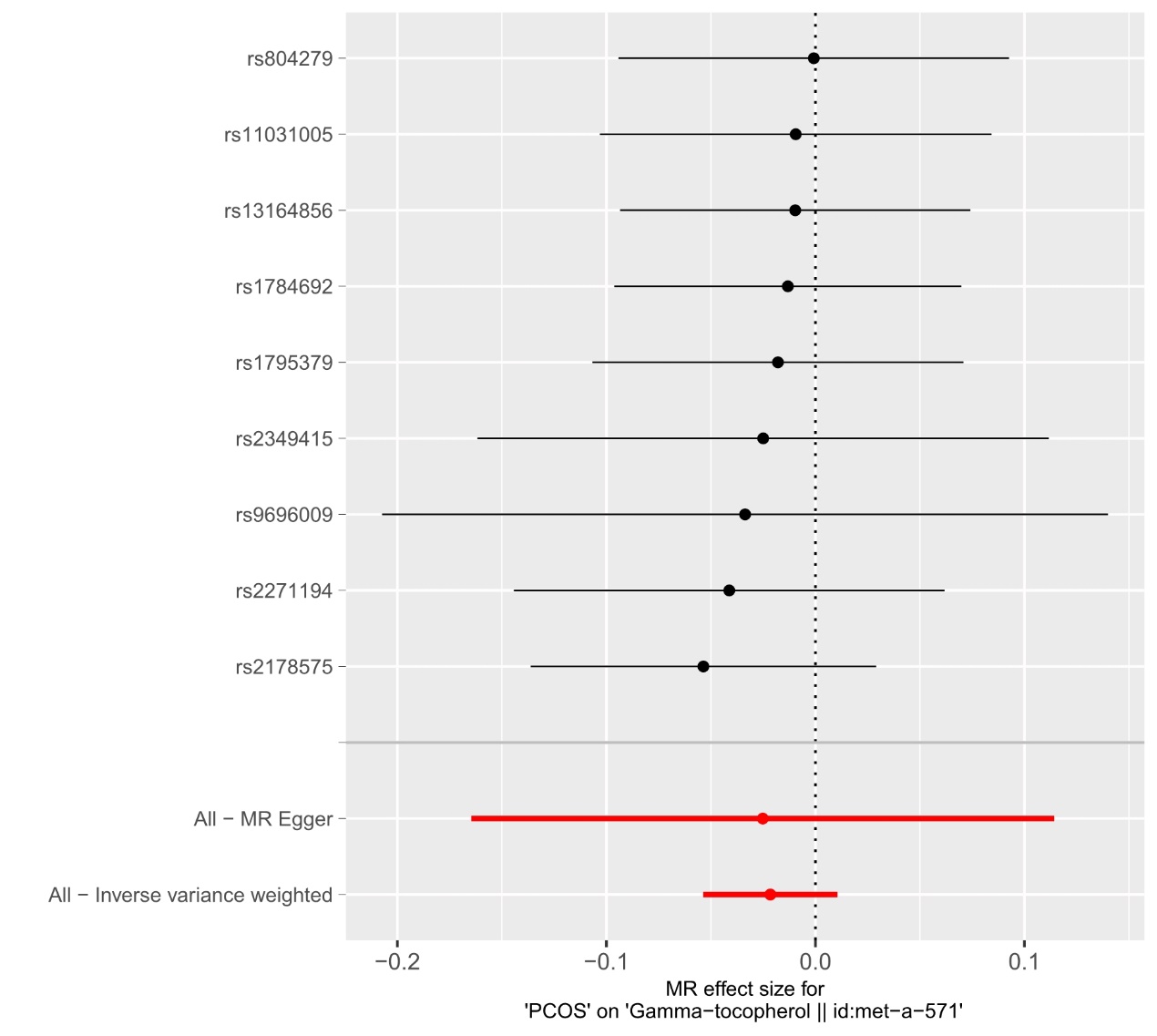


PCOS, Polycystic ovary syndrome.

Supplement: Supplementary file 14 — Supplementary Material 14 [file 12920_2023_1581_MOESM14_ESM.docx]

Figure S25. MR effect size for PCOS on ascorbate


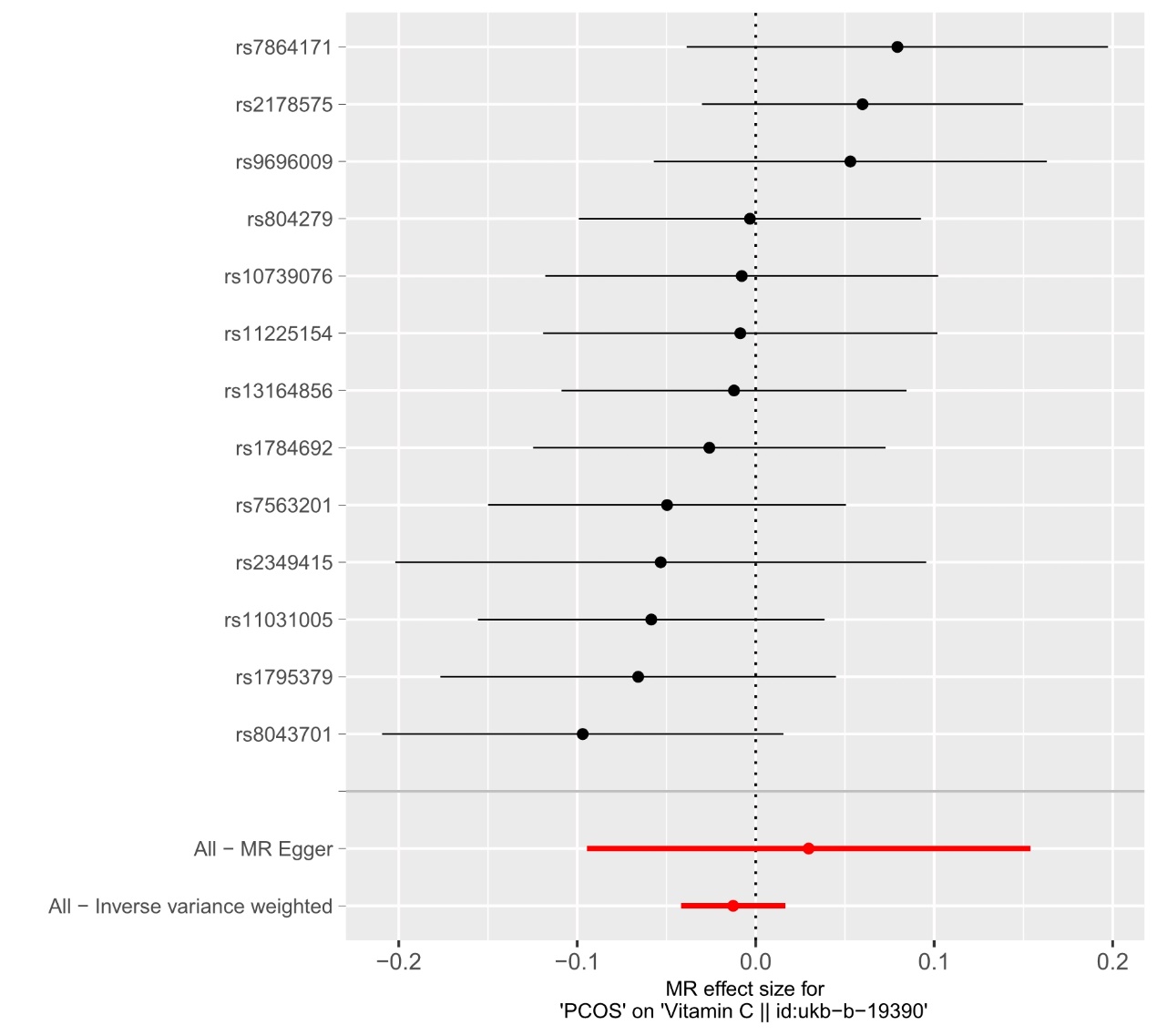


PCOS, Polycystic ovary syndrome.

Supplement: Supplementary file 18 — Supplementary Material 18 [file 12920_2023_1581_MOESM18_ESM.docx]

Figure S26. scatter plot of the MR analysis of PCOS on ascorbate


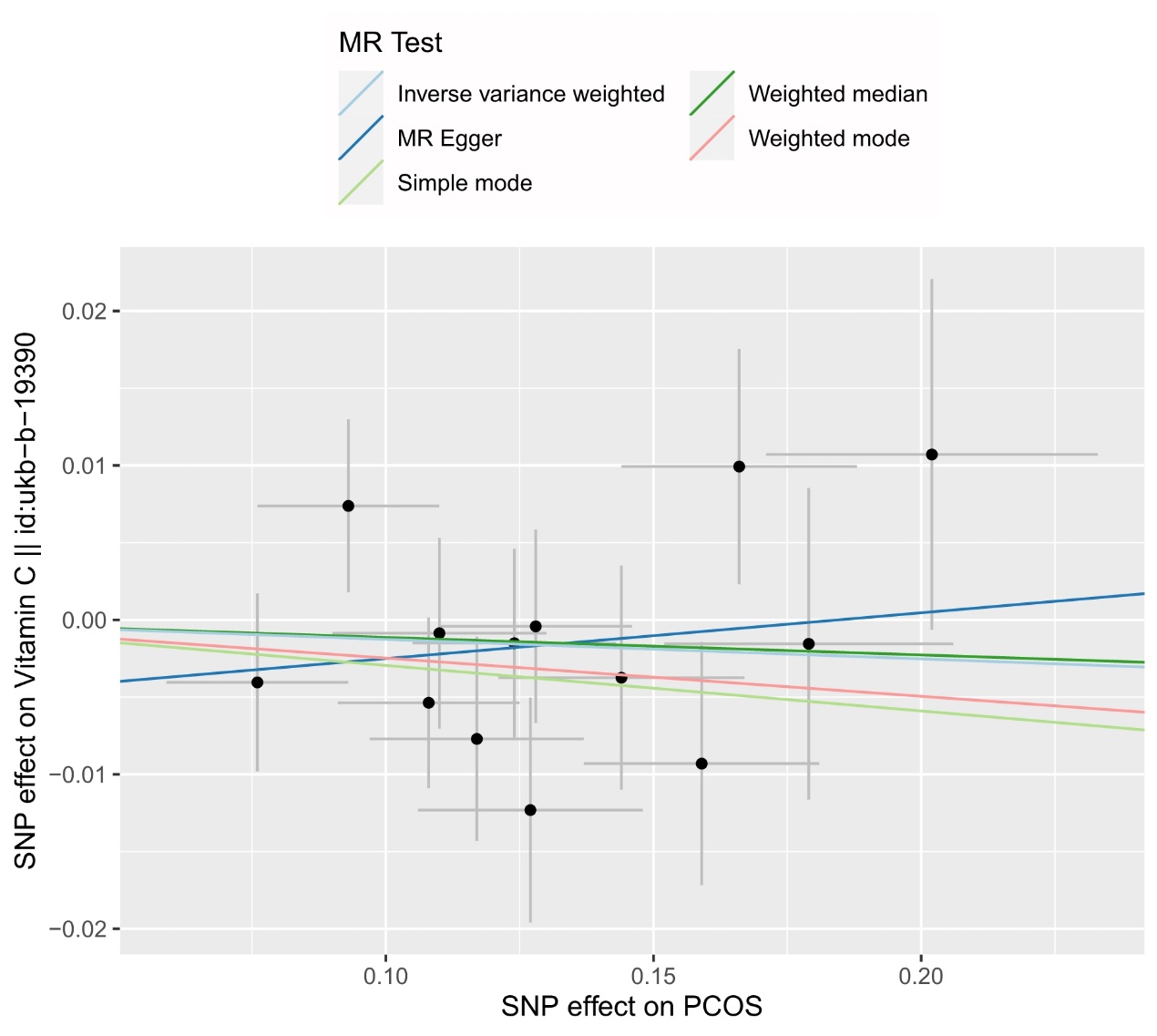


PCOS, Polycystic ovary syndrome.

Supplement: Supplementary file 19 — Supplementary Material 19 [file 12920_2023_1581_MOESM19_ESM.docx]

Figure S28. funnel plot of the MR analysis of PCOS on ascorbate


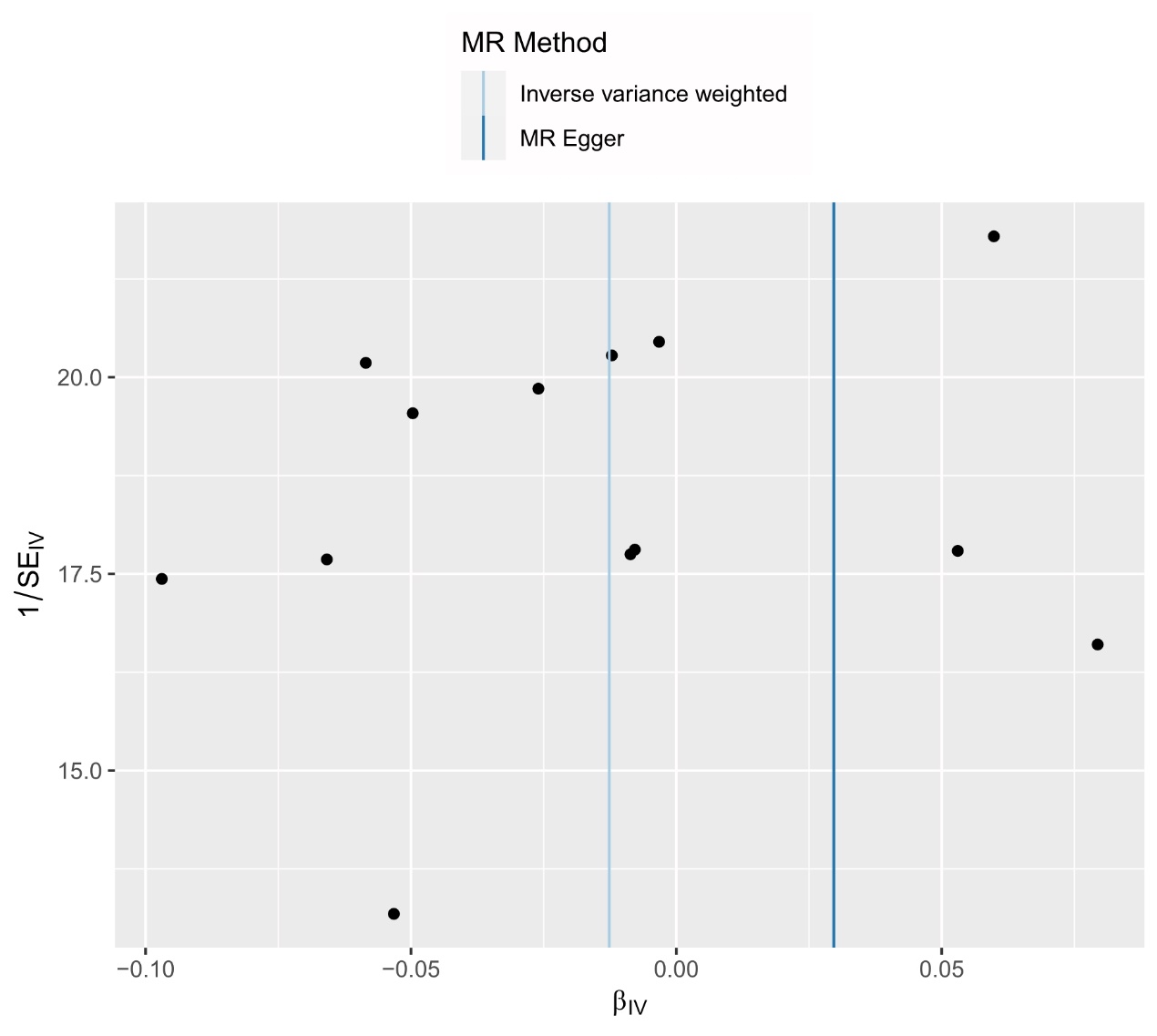


PCOS, Polycystic ovary syndrome.

Supplement: Supplementary file 21 — Supplementary Material 21 [file 12920_2023_1581_MOESM21_ESM.docx]

Figure S29. MR effect size for PCOS on retinol


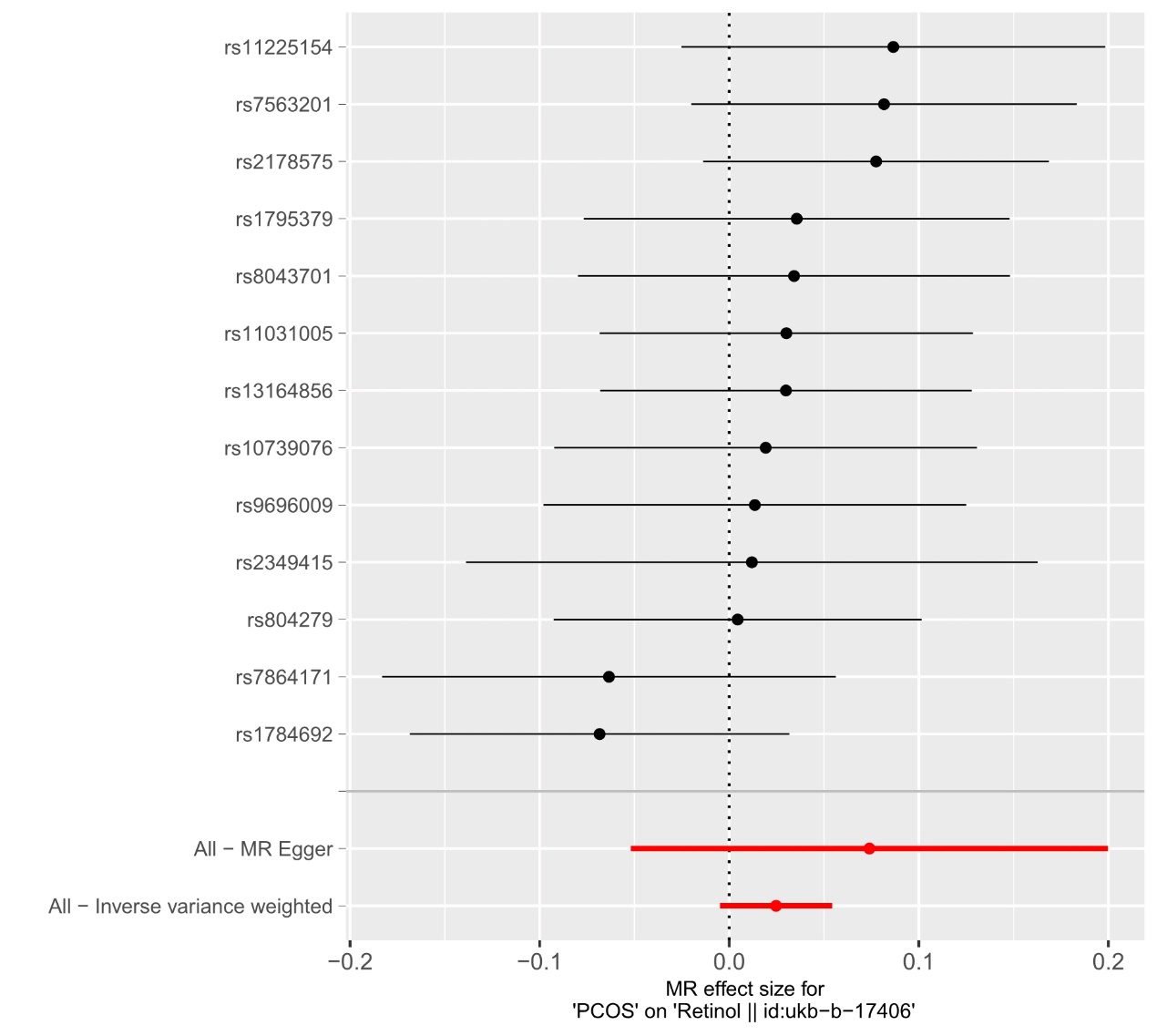


PCOS, Polycystic ovary syndrome.

Supplement: Supplementary file 22 — Supplementary Material 22 [file 12920_2023_1581_MOESM22_ESM.docx]

Figure S30. scatter plot of the MR analysis of PCOS on retinol


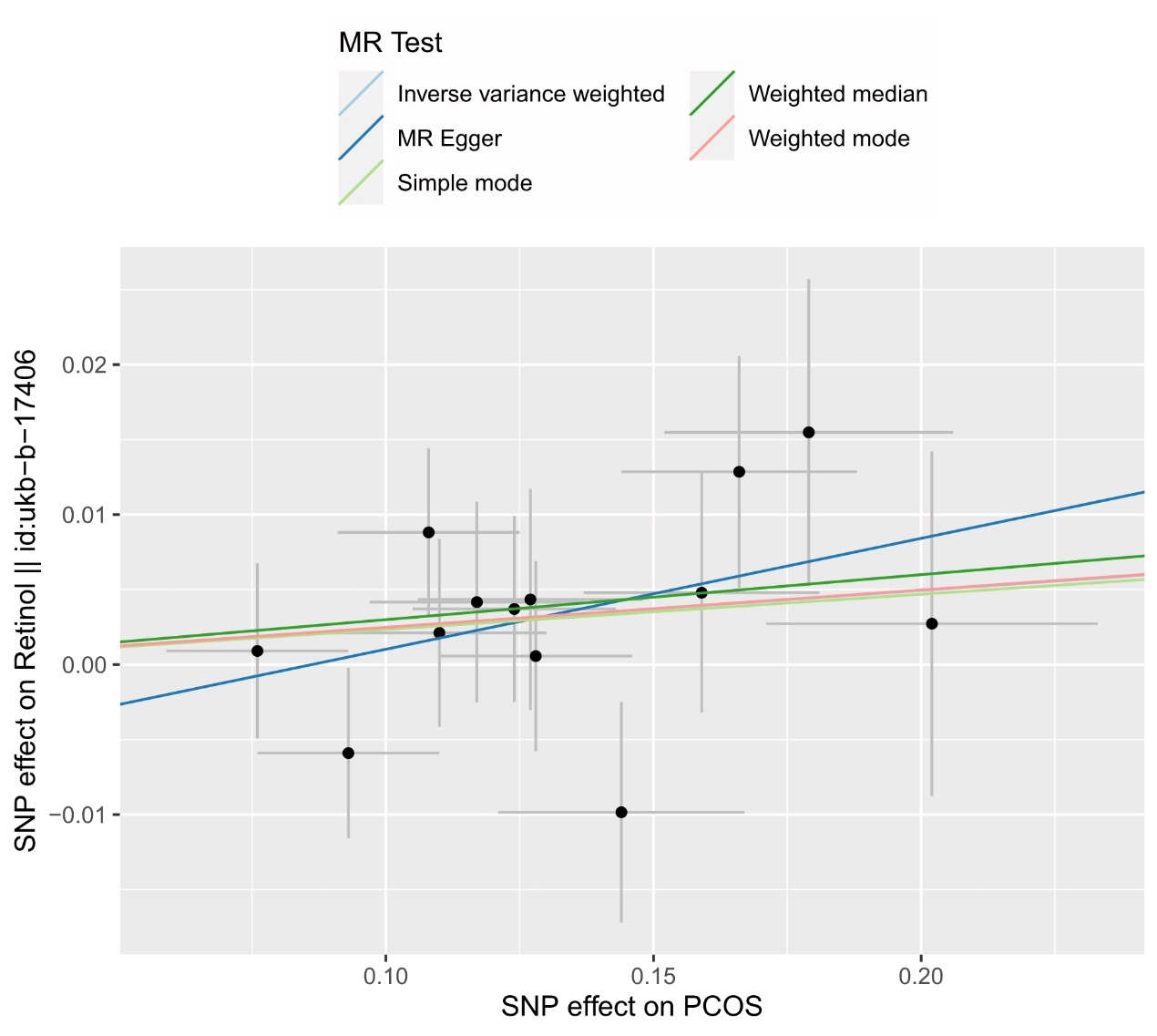


PCOS, Polycystic ovary syndrome.

Supplement: Supplementary file 24 — Supplementary Material 24 [file 12920_2023_1581_MOESM24_ESM.docx]

Figure S31. leave-one-out regression analysis of PCOS on retinol


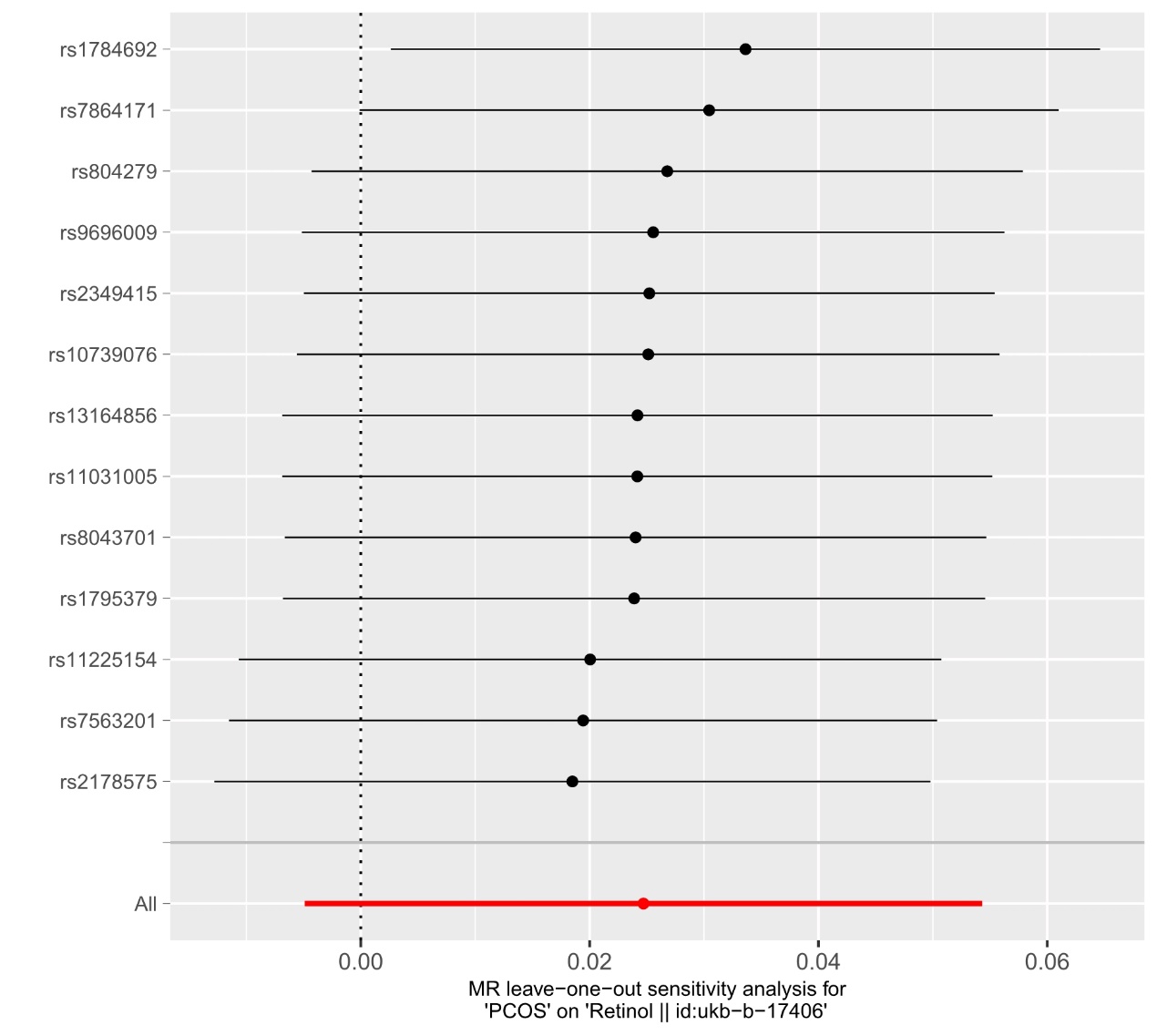


PCOS, Polycystic ovary syndrome.

Supplement: Supplementary file 25 — Supplementary Material 25 [file 12920_2023_1581_MOESM25_ESM.docx]

Figure S32. funnel plot of the MR analysis of PCOS on retinol


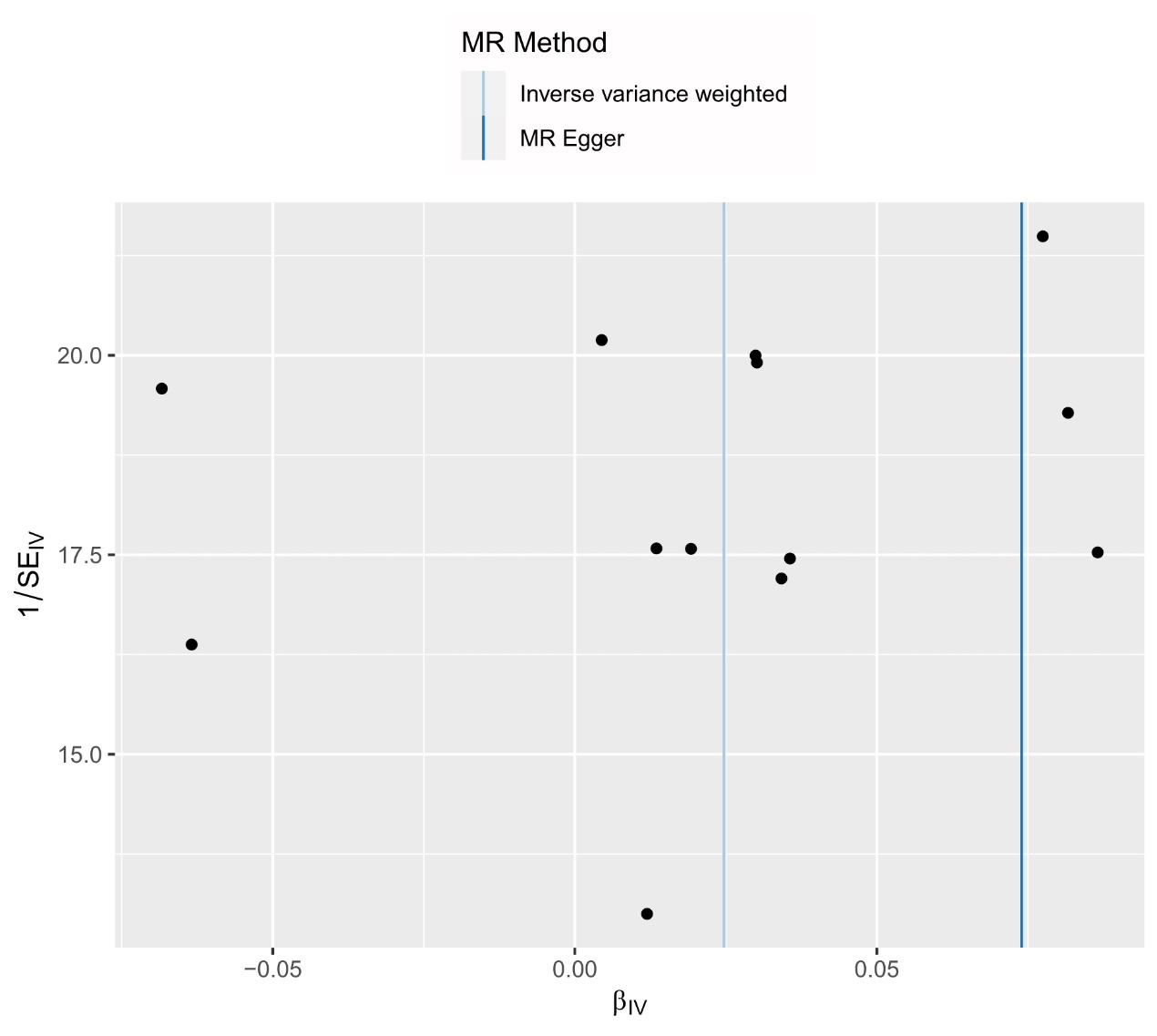


PCOS, Polycystic ovary syndrome.

Supplement: Supplementary file 26 — Supplementary Material 26 [file 12920_2023_1581_MOESM26_ESM.docx]

Figure S33. MR effect size for PCOS on albumin


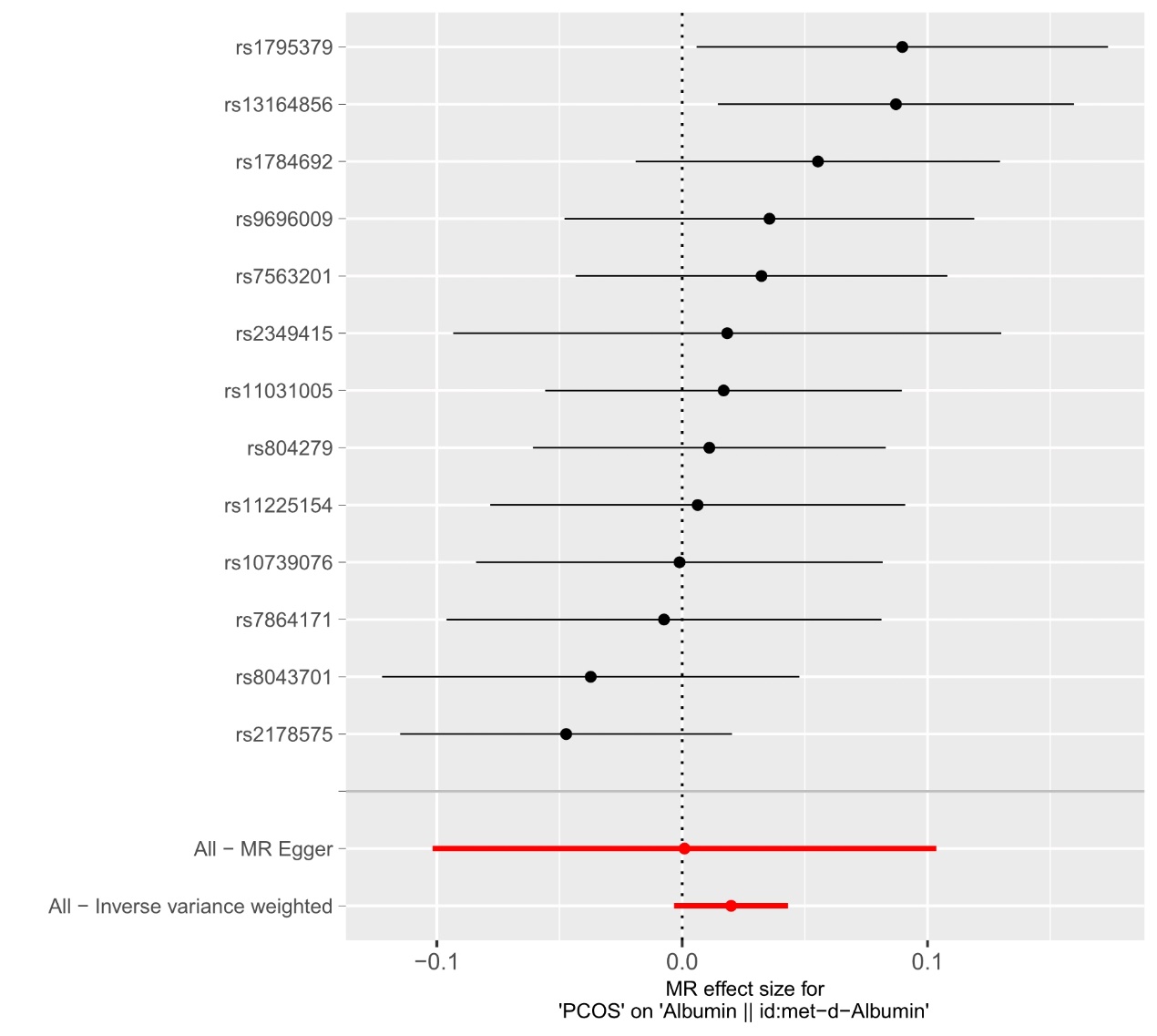


PCOS, Polycystic ovary syndrome.

Supplement: Supplementary file 27 — Supplementary Material 27 [file 12920_2023_1581_MOESM27_ESM.docx]

Figure S34. scatter plot of the MR analysis of PCOS on albumin


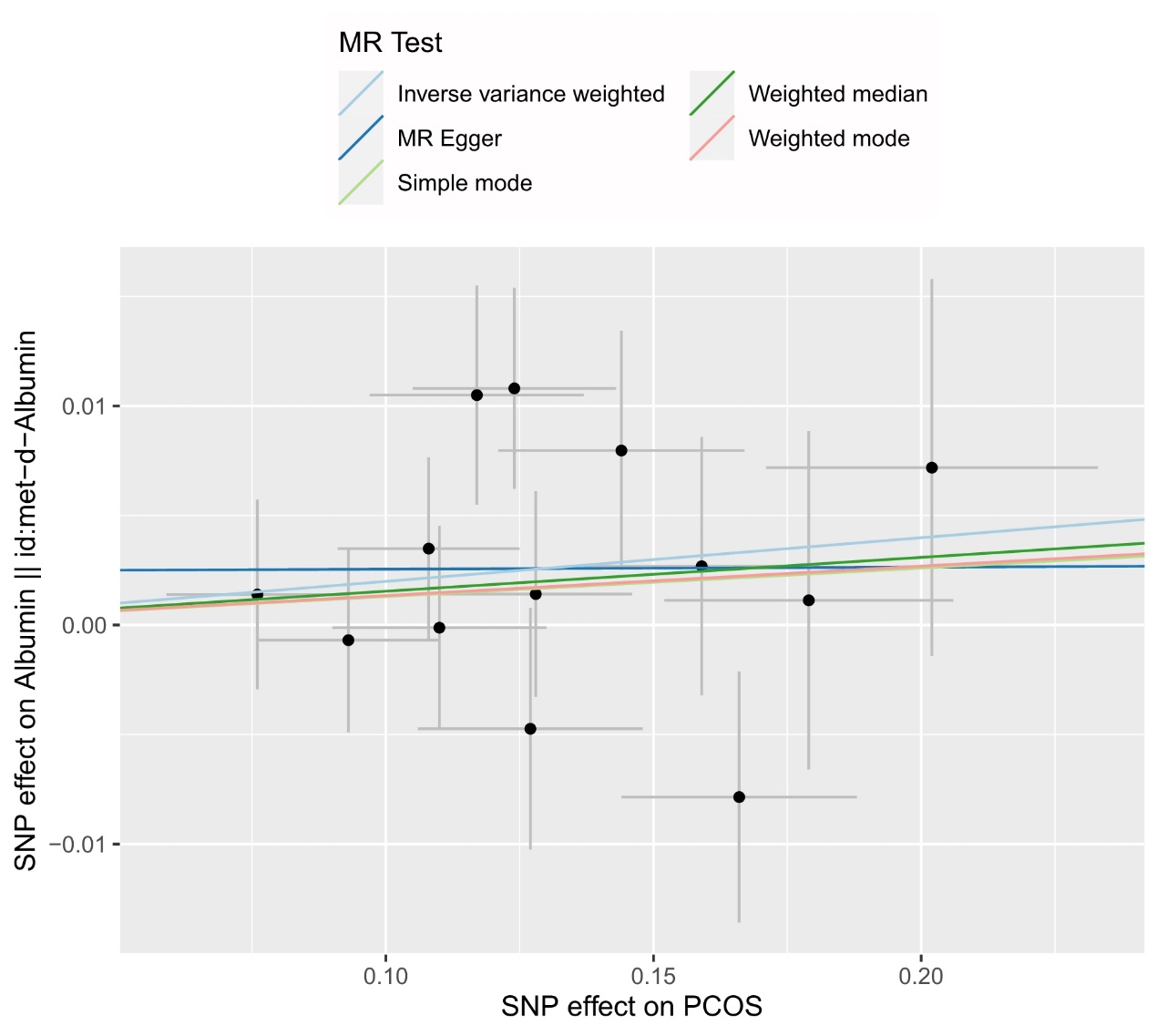


PCOS, Polycystic ovary syndrome.

Supplement: Supplementary file 28 — Supplementary Material 28 [file 12920_2023_1581_MOESM28_ESM.docx]

Figure S35. leave-one-out regression analysis of PCOS on albumin


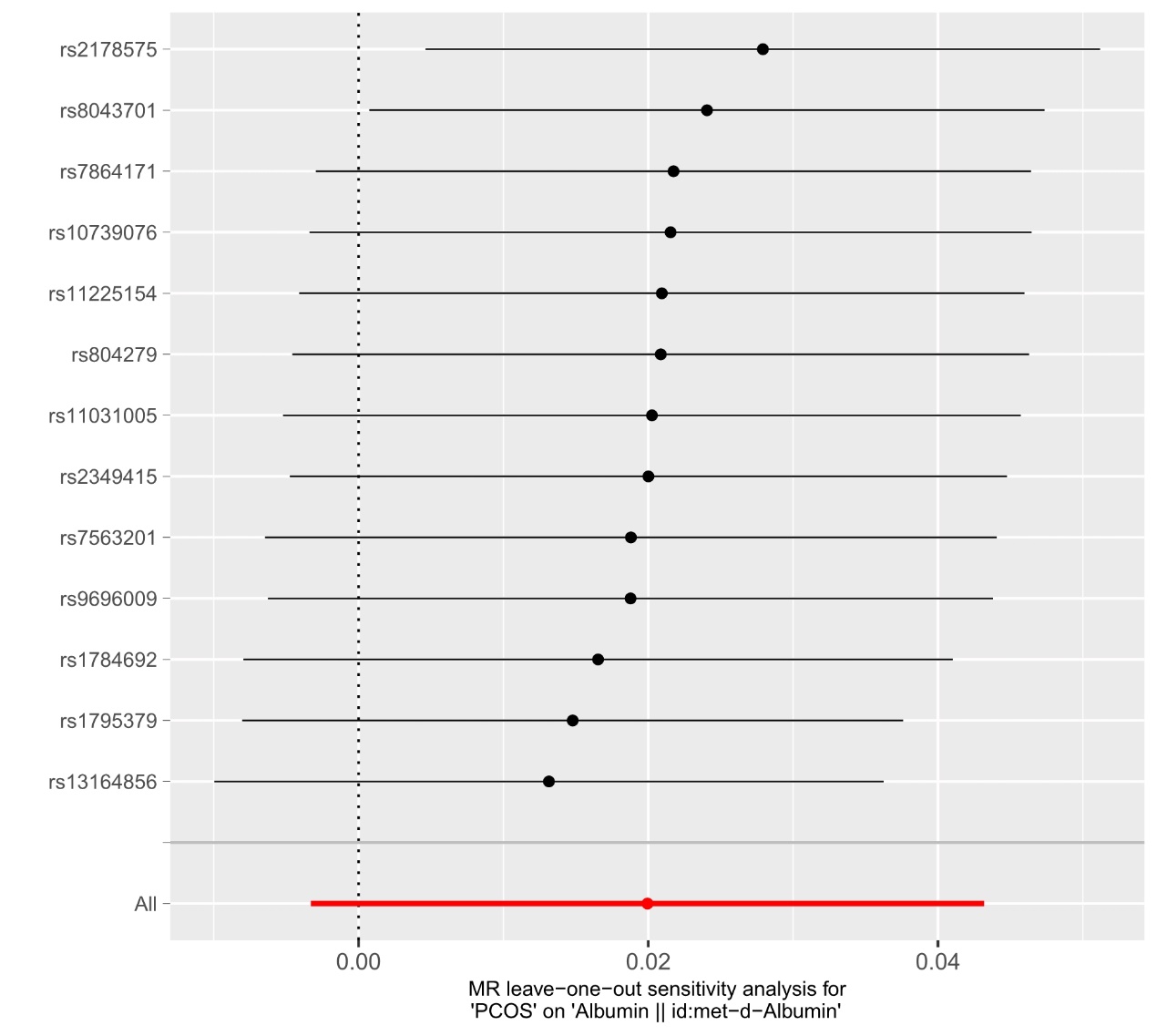


PCOS, Polycystic ovary syndrome.

Supplement: Supplementary file 29 — Supplementary Material 29 [file 12920_2023_1581_MOESM29_ESM.docx]

Figure S36. funnel plot of the MR analysis of PCOS on albumin


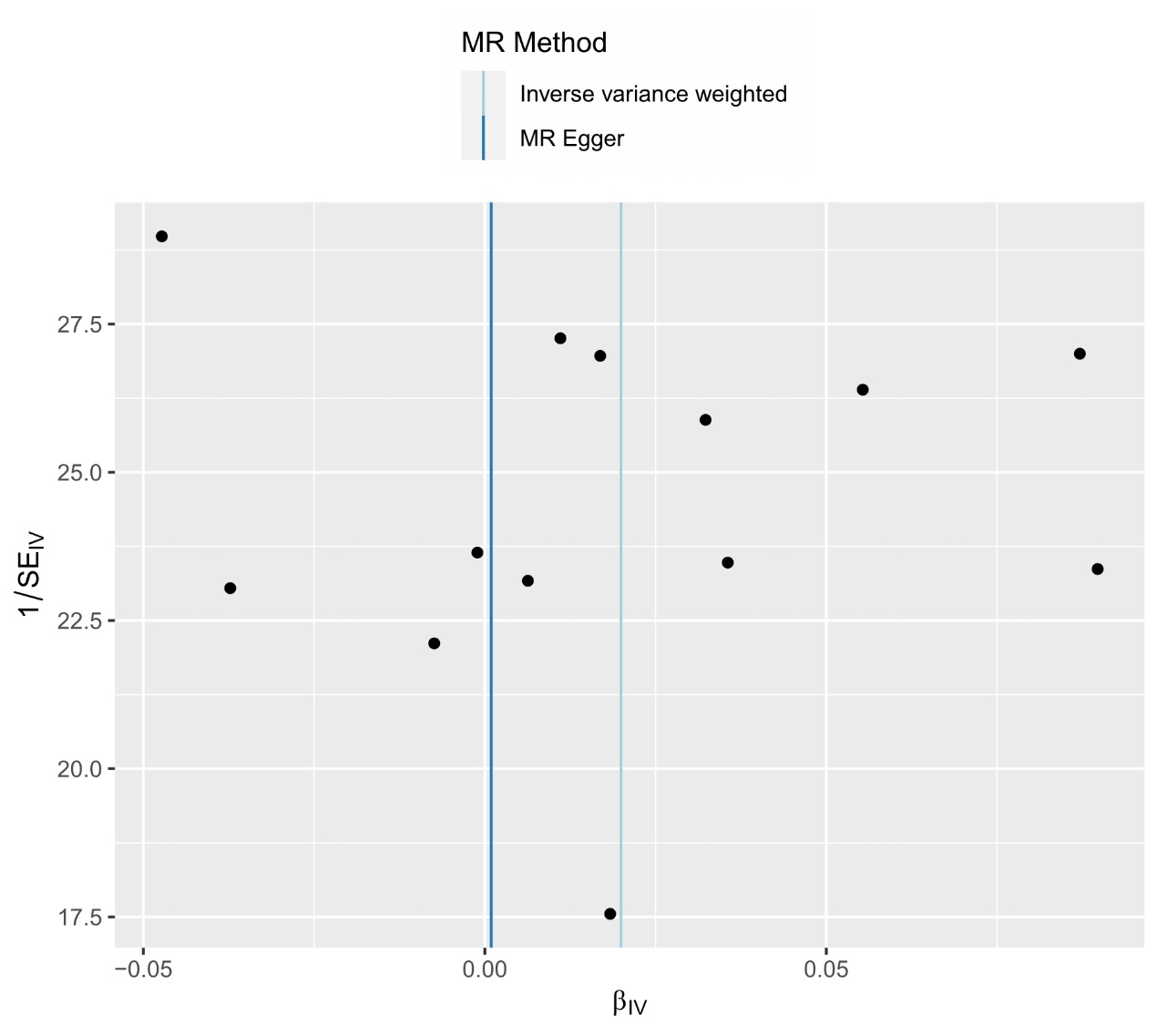


PCOS, Polycystic ovary syndrome.

Supplement: Supplementary file 30 — Supplementary Material 30 [file 12920_2023_1581_MOESM30_ESM.docx]

Figure S37. MR effect size for PCOS on TBIL


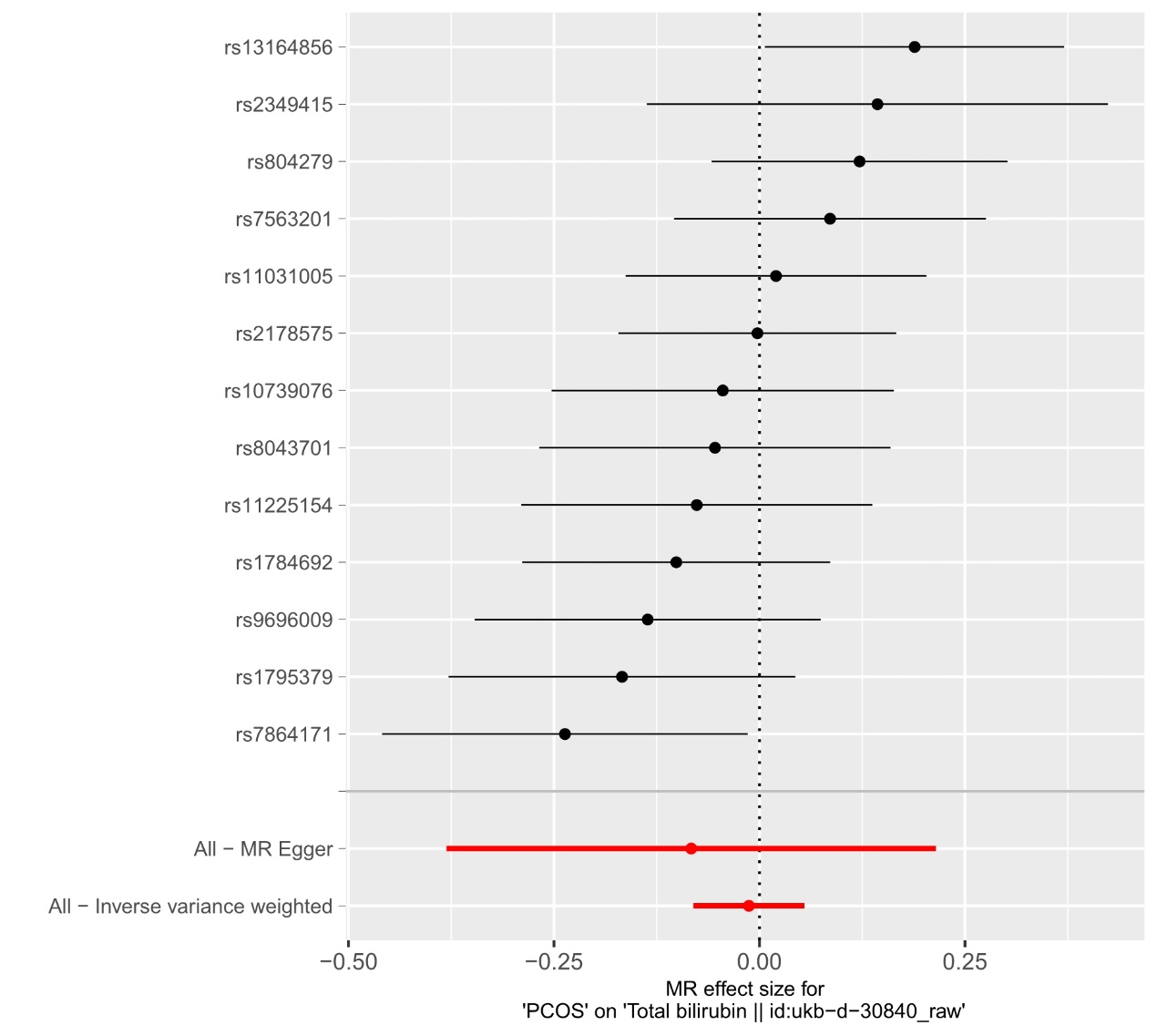


PCOS, Polycystic ovary syndrome; TBIL, total bilirubin.

Supplement: Supplementary file 31 — Supplementary Material 31 [file 12920_2023_1581_MOESM31_ESM.docx]

Figure S9. MR effect size for PCOS on CAT


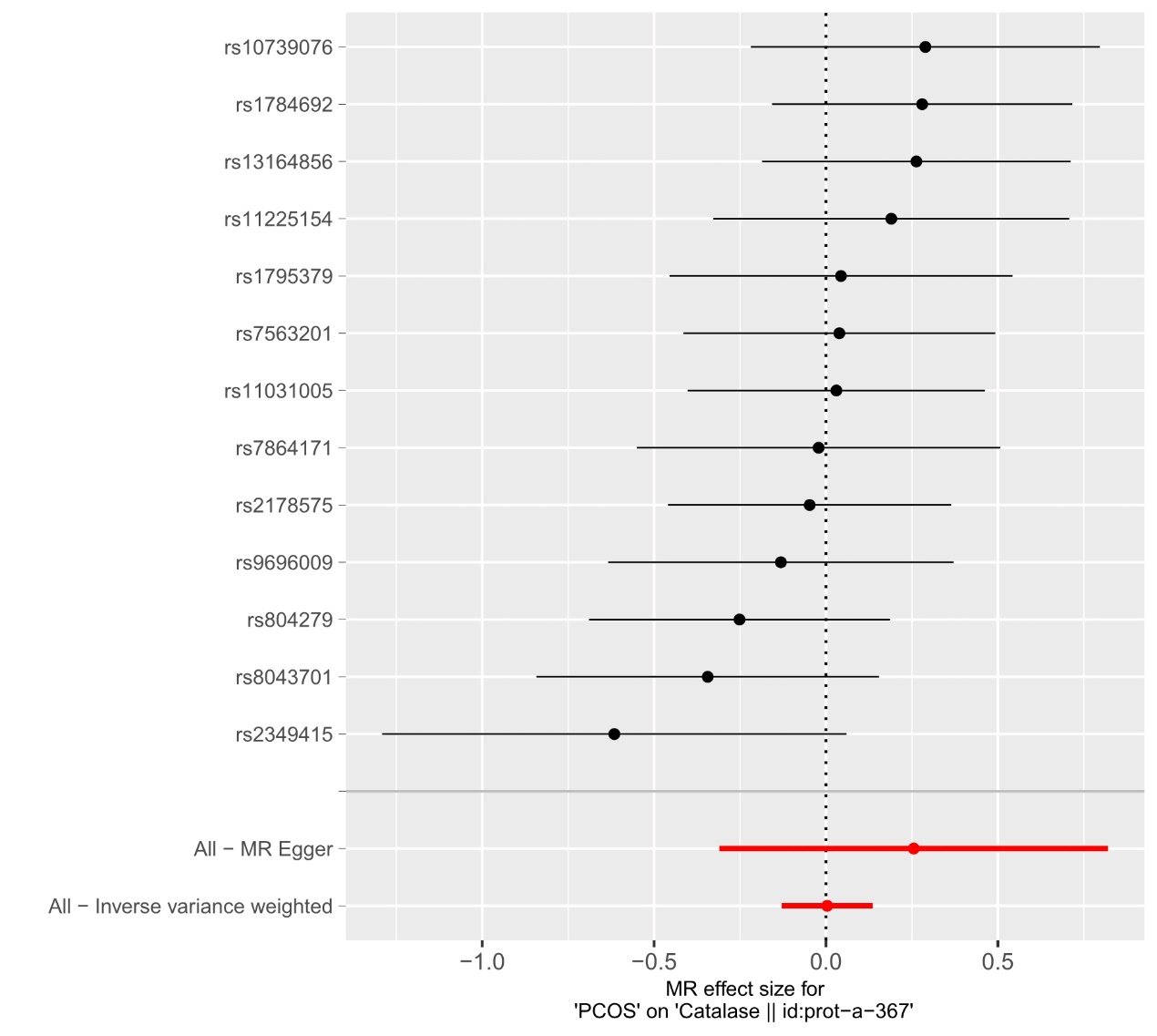


PCOS, Polycystic ovary syndrome; CAT, catalase.

Supplement: Supplementary file 40 — Supplementary Material 40 [file 12920_2023_1581_MOESM40_ESM.docx]
