# Supplementary material for: Evidence for causal effects of polycystic ovary syndrome on oxidative stress: a two-sample mendelian randomisation study
Source: BMC Med Genomics. 2023 Jun 19;16:141. doi: 10.1186/s12920-023-01581-0 (PMC10278295; doi:10.1186/s12920-023-01581-0)
Supplement: Supplementary file 20 — Supplementary Material 20 [file 12920_2023_1581_MOESM20_ESM.docx]

Figure S27. leave-one-out regression analysis of PCOS on ascorbate


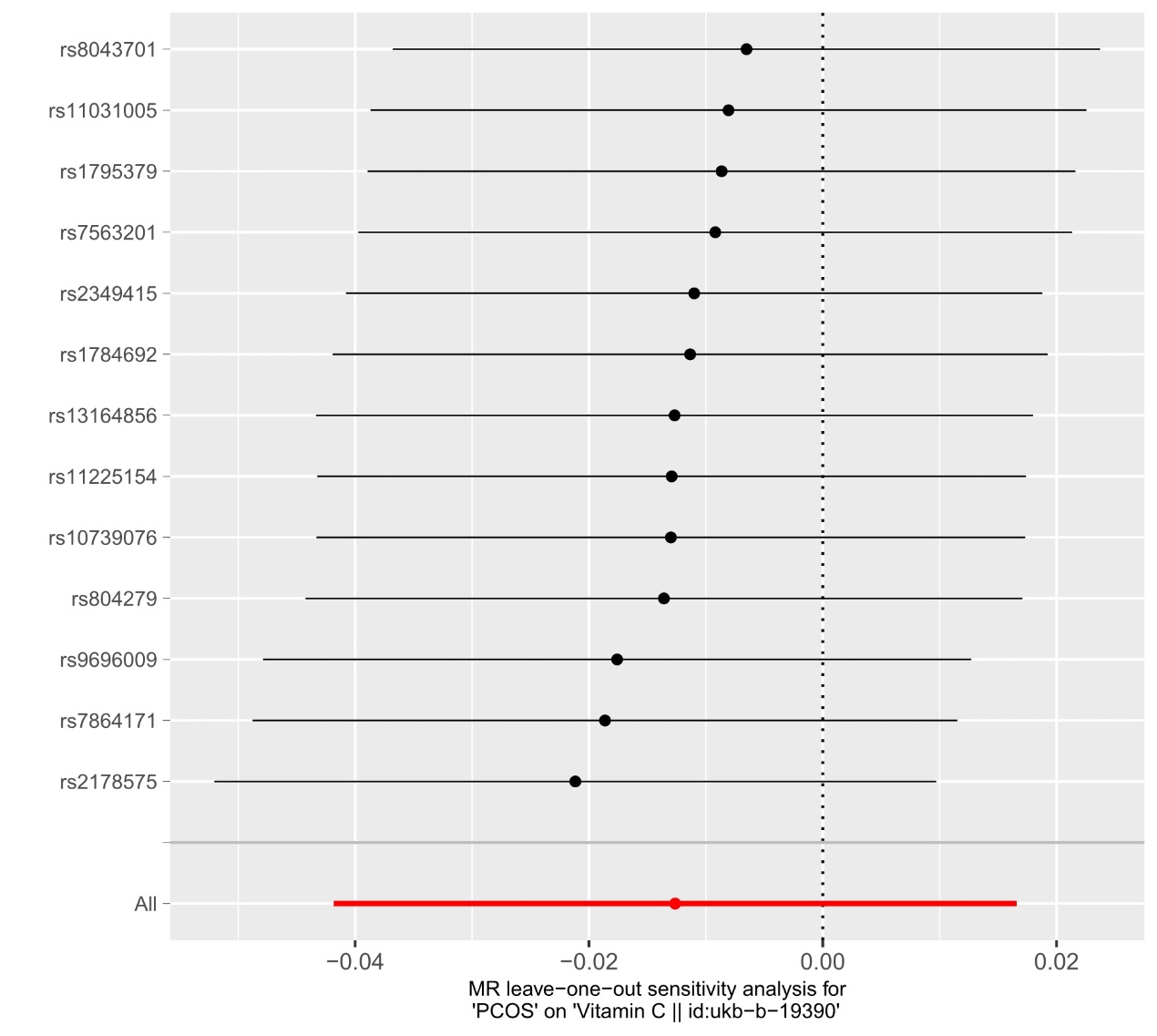


PCOS, Polycystic ovary syndrome.
