# Supplementary material for: Evidence for causal effects of polycystic ovary syndrome on oxidative stress: a two-sample mendelian randomisation study
Source: BMC Med Genomics. 2023 Jun 19;16:141. doi: 10.1186/s12920-023-01581-0 (PMC10278295; doi:10.1186/s12920-023-01581-0)
Supplement: Supplementary file 42 — Supplementary Material 42 [file 12920_2023_1581_MOESM42_ESM.docx]

| Methods | IVs (n SNPs) | Beta | SE | P | OR | 95%CI |
| --- | --- | --- | --- | --- | --- | --- |
| MR Egger | 13 | 0.357 | 0.322 | 0.291 | 1.428 | 0.760，2.683 |
| Weighted median | 13 | -0.019 | 0.091 | 0.835 | 0.981 | 0.821，1.173 |
| Inverse variance weighted | 13 | 0.007 | 0.076 | 0.932 | 1.007 | 0.867，1.169 |
| Simple mode | 13 | -0.121 | 0.145 | 0.420 | 0.886 | 0.667，1.177 |
| Weighted mode | 13 | -0.061 | 0.138 | 0.665 | 0.941 | 0.717，1.233 |

Table S1 Causal association between PCOS and GST (ieu ID: prot-a-1283). SNP, Single Nucleotide polymorphisms; IVs, instrumental variables; OR, Odds ratio; CI, confidence interval; SE, standard error; n, number
