# Supplementary material for: Evidence for causal effects of polycystic ovary syndrome on oxidative stress: a two-sample mendelian randomisation study
Source: BMC Med Genomics. 2023 Jun 19;16:141. doi: 10.1186/s12920-023-01581-0 (PMC10278295; doi:10.1186/s12920-023-01581-0)
Supplement: Supplementary file 43 — Supplementary Material 43 [file 12920_2023_1581_MOESM43_ESM.docx]

| Methods | IVs (n SNPs) | Beta | SE | P | OR | 95%CI |
| --- | --- | --- | --- | --- | --- | --- |
| MR Egger | 13 | -0.083 | 0.152 | 0.595 | 0.920 | 0.683，1.239 |
| Weighted median | 13 | -0.023 | 0.042 | 0.589 | 0.977 | 0.900，1.062 |
| Inverse variance weighted | 13 | -0.013 | 0.034 | 0.709 | 0.987 | 0.923，1.056 |
| Simple mode | 13 | -0.061 | 0.079 | 0.455 | 0.941 | 0.806，1.098 |
| Weighted mode | 13 | -0.044 | 0.071 | 0.545 | 0.956 | 0.831，1.100 |

Table S10 Causal association between PCOS and Total bilirubin (ieu ID: ukb-d-30840_raw). SNP, Single Nucleotide polymorphisms; IVs, instrumental variables; OR, Odds ratio; CI, confidence interval; SE, standard error; n, number
