# Supplementary material for: Evidence for causal effects of polycystic ovary syndrome on oxidative stress: a two-sample mendelian randomisation study
Source: BMC Med Genomics. 2023 Jun 19;16:141. doi: 10.1186/s12920-023-01581-0 (PMC10278295; doi:10.1186/s12920-023-01581-0)
Supplement: Supplementary file 44 — Supplementary Material 44 [file 12920_2023_1581_MOESM44_ESM.docx]

| Methods | IVs (n SNPs) | Beta | SE | P | OR | 95%CI |
| --- | --- | --- | --- | --- | --- | --- |
| MR Egger | 13 | -0.299 | 0.404 | 0.474 | 0.741 | 0.336，1.636 |
| Weighted median | 13 | 0.026 | 0.106 | 0.809 | 1.026 | 0.834，1.262 |
| Inverse variance weighted | 13 | 0.014 | 0.093 | 0.879 | 1.014 | 0.845，1.218 |
| Simple mode | 13 | -0.079 | 0.225 | 0.730 | 0.924 | 0.595，1.435 |
| Weighted mode | 13 | -0.151 | 0.263 | 0.578 | 0.860 | 0.513，1.441 |

Table S2 Causal association between PCOS and GPX (ieu ID: prot-a-1265). SNP, Single Nucleotide polymorphisms; IVs, instrumental variables; OR, Odds ratio; CI, confidence interval; SE, standard error; n, number
