# Supplementary material for: Evidence for causal effects of polycystic ovary syndrome on oxidative stress: a two-sample mendelian randomisation study
Source: BMC Med Genomics. 2023 Jun 19;16:141. doi: 10.1186/s12920-023-01581-0 (PMC10278295; doi:10.1186/s12920-023-01581-0)
Supplement: Supplementary file 45 — Supplementary Material 45 [file 12920_2023_1581_MOESM45_ESM.docx]

| Methods | IVs (n SNPs) | Beta | SE | P | OR | 95%CI |
| --- | --- | --- | --- | --- | --- | --- |
| MR Egger | 13 | -0.255 | 0.288 | 0.395 | 1.291 | 0.733，2.272 |
| Weighted median | 13 | 0.032 | 0.092 | 0.731 | 1.032 | 0.861，1.237 |
| Inverse variance weighted | 13 | 0.004 | 0.068 | 0.958 | 1.004 | 0.879，1.146 |
| Simple mode | 13 | 0.029 | 0.156 | 0.856 | 1.029 | 0.759，1.396 |
| Weighted mode | 13 | 0.022 | 0.150 | 0.885 | 1.022 | 0.762，1.372 |

Table S3 Causal association between PCOS and CAT (ieu ID: prot-a-367). SNP, Single Nucleotide polymorphisms; IVs, instrumental variables; OR, Odds ratio; CI, confidence interval; SE, standard error; n, number
