# Supplementary material for: Evidence for causal effects of polycystic ovary syndrome on oxidative stress: a two-sample mendelian randomisation study
Source: BMC Med Genomics. 2023 Jun 19;16:141. doi: 10.1186/s12920-023-01581-0 (PMC10278295; doi:10.1186/s12920-023-01581-0)
Supplement: Supplementary file 46 — Supplementary Material 46 [file 12920_2023_1581_MOESM46_ESM.docx]

| Methods | IVs (n SNPs) | Beta | SE | P | OR | 95%CI |
| --- | --- | --- | --- | --- | --- | --- |
| MR Egger | 13 | -1.023 | 3.265 | 0.760 | 0.359 | 0.001， 216.344 |
| Weighted median | 13 | 0.408 | 0.684 | 0.550 | 1.505 | 0.394， 5.750 |
| Inverse variance weighted | 13 | 1.105 | 0.748 | 0.140 | 3.018 | 0.696， 13.087 |
| Simple mode | 13 | 0.363 | 1.021 | 0.728 | 1.437 | 0.194， 10.621 |
| Weighted mode | 13 | 0.385 | 1.056 | 0.721 | 1.470 | 0.186， 11.641 |

Table S4 Causal association between PCOS and UA (ieu ID: ukb-d-30880_raw). SNP, Single Nucleotide polymorphisms; IVs, instrumental variables; OR, Odds ratio; CI, confidence interval; SE, standard error; n, number
