# Supplementary material for: Evidence for causal effects of polycystic ovary syndrome on oxidative stress: a two-sample mendelian randomisation study
Source: BMC Med Genomics. 2023 Jun 19;16:141. doi: 10.1186/s12920-023-01581-0 (PMC10278295; doi:10.1186/s12920-023-01581-0)
Supplement: Supplementary file 47 — Supplementary Material 47 [file 12920_2023_1581_MOESM47_ESM.docx]

| Methods | IVs (n SNPs) | Beta | SE | P | OR | 95%CI |
| --- | --- | --- | --- | --- | --- | --- |
| MR Egger | 7 | 0.145 | 0.384 | 0.722 | 1.156 | 0.544，2.455 |
| Weighted median | 7 | -0.050 | 0.125 | 0.687 | 0.951 | 0.744，1.215 |
| Inverse variance weighted | 7 | -0.008 | 0.096 | 0.934 | 0.992 | 0.821，1.198 |
| Simple mode | 7 | -0.083 | 0.184 | 0.666 | 0.920 | 0.642，1.319 |
| Weighted mode | 7 | -0.068 | 0.182 | 0.722 | 0.934 | 0.654，1.335 |

Table S5 Causal association between PCOS and Zinc (ieu ID: ieu-a-1079). SNP, Single Nucleotide polymorphisms; IVs, instrumental variables; OR, Odds ratio; CI, confidence interval; SE, standard error; n, number
