# Supplementary material for: Evidence for causal effects of polycystic ovary syndrome on oxidative stress: a two-sample mendelian randomisation study
Source: BMC Med Genomics. 2023 Jun 19;16:141. doi: 10.1186/s12920-023-01581-0 (PMC10278295; doi:10.1186/s12920-023-01581-0)
Supplement: Supplementary file 48 — Supplementary Material 48 [file 12920_2023_1581_MOESM48_ESM.docx]

| Methods | IVs (n SNPs) | Beta | SE | P | OR | 95%CI |
| --- | --- | --- | --- | --- | --- | --- |
| MR Egger | 9 | -0.025 | 0.071 | 0.734 | 0.975 | 0.848，1.121 |
| Weighted median | 9 | -0.015 | 0.020 | 0.475 | 0.986 | 0.947，1.026 |
| Inverse variance weighted | 9 | -0.022 | 0.016 | 0.188 | 0.979 | 0.948，1.011 |
| Simple mode | 9 | -0.012 | 0.032 | 0.712 | 0.988 | 0.928，1.051 |
| Weighted mode | 9 | -0.012 | 0.026 | 0.669 | 0.988 | 0.939，1.040 |

Table S6 Causal association between PCOS and alpha-tocopherol (ieu ID: met-a-571). SNP, Single Nucleotide polymorphisms; IVs, instrumental variables; OR, Odds ratio; CI, confidence interval; SE, standard error; n, number
