# Supplementary material for: Evidence for causal effects of polycystic ovary syndrome on oxidative stress: a two-sample mendelian randomisation study
Source: BMC Med Genomics. 2023 Jun 19;16:141. doi: 10.1186/s12920-023-01581-0 (PMC10278295; doi:10.1186/s12920-023-01581-0)
Supplement: Supplementary file 49 — Supplementary Material 49 [file 12920_2023_1581_MOESM49_ESM.docx]

| Methods | IVs (n SNPs) | Beta | SE | P | OR | 95%CI |
| --- | --- | --- | --- | --- | --- | --- |
| MR Egger | 13 | 0.030 | 0.063 | 0.649 | 1.030 | 0.910，1.166 |
| Weighted median | 13 | -0.011 | 0.020 | 0.573 | 0.989 | 0.950，1.028 |
| Inverse variance weighted | 13 | -0.013 | 0.015 | 0.397 | 0.987 | 0.959，1.017 |
| Simple mode | 13 | -0.029 | 0.034 | 0.409 | 0.971 | 0.907，1.039 |
| Weighted mode | 13 | -0.025 | 0.036 | 0.505 | 0.976 | 0.909，1.047 |

Table S7 Causal association between PCOS and Ascorbic acid (ieu ID: ukb-b-19390). SNP, Single Nucleotide polymorphisms; IVs, instrumental variables; OR, Odds ratio; CI, confidence interval; SE, standard error; n, number
