# Supplementary material for: Evidence for causal effects of polycystic ovary syndrome on oxidative stress: a two-sample mendelian randomisation study
Source: BMC Med Genomics. 2023 Jun 19;16:141. doi: 10.1186/s12920-023-01581-0 (PMC10278295; doi:10.1186/s12920-023-01581-0)
Supplement: Supplementary file 50 — Supplementary Material 50 [file 12920_2023_1581_MOESM50_ESM.docx]

| Methods | IVs (n SNPs) | Beta | SE | P | OR | 95%CI |
| --- | --- | --- | --- | --- | --- | --- |
| MR Egger | 13 | 0.074 | 0.064 | 0.274 | 1.077 | 0.949，1.221 |
| Weighted median | 13 | 0.030 | 0.020 | 0.134 | 1.030 | 0.990，1.073 |
| Inverse variance weighted | 13 | 0.025 | 0.015 | 0.102 | 1.025 | 0.995，1.056 |
| Simple mode | 13 | 0.023 | 0.034 | 0.506 | 1.024 | 0.958，1.094 |
| Weighted mode | 13 | 0.025 | 0.034 | 0.480 | 1.025 | 0.959，1.096 |

Table S8 Causal association between PCOS and retinol (ieu ID: ukb-b-17406). SNP, Single Nucleotide polymorphisms; IVs, instrumental variables; OR, Odds ratio; CI, confidence interval; SE, standard error; n, number
