# Supplementary material for: Evidence for causal effects of polycystic ovary syndrome on oxidative stress: a two-sample mendelian randomisation study
Source: BMC Med Genomics. 2023 Jun 19;16:141. doi: 10.1186/s12920-023-01581-0 (PMC10278295; doi:10.1186/s12920-023-01581-0)
Supplement: Supplementary file 51 — Supplementary Material 51 [file 12920_2023_1581_MOESM51_ESM.docx]

| Methods | IVs (n SNPs) | Beta | SE | P | OR | 95%CI |
| --- | --- | --- | --- | --- | --- | --- |
| MR Egger | 13 | 0.001 | 0.052 | 0.986 | 1.001 | 0.903， 1.109 |
| Weighted median | 13 | 0.015 | 0.016 | 0.342 | 1.016 | 0.984， 1.048 |
| Inverse variance weighted | 13 | 0.020 | 0.012 | 0.093 | 1.020 | 0.997， 1.044 |
| Simple mode | 13 | 0.013 | 0.029 | 0.659 | 1.013 | 0.958， 1.072 |
| Weighted mode | 13 | 0.013 | 0.029 | 0.650 | 1.013 | 0.958， 1.073 |

Table S9 Causal association between PCOS and Albumin (ieu ID: met-d- Albumin). SNP, Single Nucleotide polymorphisms; IVs, instrumental variables; OR, Odds ratio; CI, confidence interval; SE, standard error; n, number
